# The Gut Microbiome of Two Wild Bumble Bee Species Native of South America: *Bombus pauloensis* and *Bombus bellicosus*

**DOI:** 10.1007/s00248-024-02430-y

**Published:** 2024-09-28

**Authors:** Gregorio Fernandez de Landa, Daniele Alberoni, Chiara Braglia, Loredana Baffoni, Mateo Fernandez de Landa, Pablo Damian Revainera, Silvina Quintana, Francisco Zumpano, Matias Daniel Maggi, Diana Di Gioia

**Affiliations:** 1 Facultad de Ciencias Exactas y Naturales, Centro Científico Tecnológico Mar del Plata, CONICET, Centro de Asociación Simple CIC PBA, Instituto de Investigaciones en Producción Sanidad y Ambiente (IIPROSAM), Universidad Nacional de Mar del Plata, Mar del Plata, Argentina; 2 Centro de Investigaciones en Abejas Sociales, Facultad de Ciencias Exactas y Naturales, Universidad Nacional de Mar del Plata, Mar del Plata, Argentina; 3 Dipartimento di Scienze e Tecnologie Agro-Alimentari, Università di Bologna, Viale Fanin 44, 40127 Bologna, Italy; 4 Facultad de Ciencias Exactas y Naturales, Instituto de Investigaciones Marinas y Costeras (IIMyC), Universidad Nacional de Mar del Plata-CONICET, Funes 3350, (7600) Mar del Plata, Argentina

**Keywords:** Bumble bees, *Bombus pauloensis*, *Bombus bellicosus*, Gut microbiome, *Nosema ceranae*

## Abstract

South America is populated by a wide range of bumble bee species that represent an important source of biodiversity, supporting pollination services in natural and agricultural ecosystems. These pollinators provide unique specific microbial niches, populated by a wide number of microorganisms such as symbionts, environmental opportunistic bacteria, and pathogens. Recently, it was demonstrated how microbial populations are shaped by trophic resources and environmental conditions but also by anthropogenic pressure, which strongly affects microbes’ functionality. This study is focused on the impact of different land uses (natural reserve, agroecosystem, and suburban) on the gut microbiome composition of two South American bumble bees, *Bombus pauloensis* and *Bombus bellicosus.* Gut microbial DNA extracted from collected bumble bees was sequenced on the Illumina MiSeq platform and correlated with land use. *Nosema ceranae* load was analyzed with qPCR and correlated with microbiome data. Significant differences in gut microbiome composition between the two wild bumble bee species were highlighted, with notable variations in α- and β-diversity across study sites. *Bombus bellicosus* showed a high abundance of *Pseudomonas*, a genus that includes environmental saprobes, and was found to be the second major taxa populating the gut microbiome, probably indicating the vulnerability of this host to environmental pollution. Pathogen analysis unveils a high prevalence of *N. ceranae*, with *B. bellicosus* showing higher susceptibility. Finally, *Gilliamella* exhibited a negative correlation with *N. ceranae*, suggesting a potential protective role of this commensal taxon. Our findings underscore the importance of considering microbial dynamics in pollinator conservation strategies, highlighting potential interactions between gut bacteria and pathogens in shaping bumble bee health.

**Supplementary Information:**

The online version contains supplementary material available at 10.1007/s00248-024-02430-y.

## Introduction

Ecosystem services consist of several benefits provided to humans by ecosystems and sometimes transformed into economic profits [52]. Pollination is considered a key ecosystem service [53] involving 75% of globally important crops [31] and also contributing to the maintenance of plant biodiversity [22]. The highest efficiency in crop pollination is achieved when wild pollinators and managed bees cohabit the same ecological niche, co-pollinating crops [32].

The *Bombus* genus (bumble bees) consists of approximately 250 known species worldwide, mostly proliferating wild, although some of them can be reared for the pollination of several crops like strawberries, peppers, and tomatoes. Within the native bee diversity present in Argentina, eight species of bumble bees were identified [1], and among these, *Bombus pauloensis* is the most abundant one [3]. Being a generalist pollinator with a well-known life cycle, in South America, *B. pauloensis* began to be reared and managed as a commercial pollinator to the same extent as *Bombus terrestris* in Europe. Another bumble bee species of relevance in Argentina is *Bombus bellicosus*, which is distributed from the North to the Patagonian region [2]. Recent studies identified a connection between climate change and the decline of the *B. bellicosus* population in Argentina. In fact, *B. bellicosus* is experiencing a significant decline in colony abundance in its native territory []^64^, together with a reduction in the area originally populated by the bumble bee itself [29]. Both *B. pauloensis and B. bellicosus* are known as eusocial bees with nesting attitude in soil cavities covered with a layer of plant debris [34, 47].

Despite their importance in agricultural and natural systems, many wild and managed bee species are suffering a sharp decrease in their population numbers [, 25,67]. Pathogen infections represent the main biohazard for bumble bees together with habitat loss due to intensive urbanization or agriculture [20, 65].

*Bombus pauloensis* can also be affected by exogenous pathogens spilled over *by* honeybees like *Nosema ceranae *[28, 18]*.* However*,* for *N. ceranae* infection, there is currently a conflict, since authors like Fernandez de Landa et al. [28] and Plischuk et al.[54] reported a high prevalence and abundance of *N. ceranae* in South American bumble bees, but, at the same time, Ngor et al. [49] and Gisder et al. [33] have demonstrated for European bumble bees that even high abundance of *N. ceranae* spores in the gut is not correlated with an actual infection.

The gut microbial communities harbored by bees can enhance both host growth and health [24, 43]. These microbes are involved in specific functions, such as food digestion and nutrient acquisition, regulation of immune responses, and defense against pathogens and parasites [, 7,71, 4]. The correct balance of the bee gut bacterial communities is critical for maintaining bee health at the individual and colony levels [58]. However, knowledge on bee gut microbial communities is not spread among all bee species, as most studies to date have focused on *A. mellifera*, and few studies on wild pollinators [39, 50]. However, the positive effects of symbiotic microorganisms on wild pollinators have been reported in recent years. For instance, Hammer et al. [35, 36] and the work by Koch et al. [43] highlighted the crucial role of gut microorganisms in the conversion and activation of nectar metabolites, providing a natural defense against parasites and enhancing the health of *B*. *terrestris*. Finally, Steffan et al. [62] detailed in their review how microorganisms benefit both solitary and social bees through pollen digestion (microbes facilitate the breakdown of the resistant outer layer of pollen grains), nutritional supplementation (bee larvae consume microbes present in fermented pollen provisions), pollen preservation (microbes alter the pollen substrate, making it less accessible to competing microbes), and mutual defense.

This study aims to fill gaps in the current knowledge about the gut microbiome composition of two bumble bee species native to South America: *B. pauloensis* and *B. bellicosus.* Furthermore, the study aims to evaluate how the prevalence and load of *N. ceranae* and land use correlate with the microbiome of both social species in Argentina.

## Materials and Methods

Some methodological details have been described in Fernandez de Landa et al. [27], which regards the same study areas although focusing on solitary bee microbiome and its correlation with pathogens and land use, and briefly summarized in Fig. S1.

### Study Areas

Three different sampling sites were selected in the Buenos Aires province before starting the study because of their different land uses and levels of anthropization: (i) an *Actinidia deliciosa*–producing farm (Santa Paula’s, 37° 56′ 0.69″ S; 57° 40′ 40.53″ W); (ii) a natural reserve (Reserva Natural Paititi, 37° 54′ 47.774″ S, 57° 48′ 44.806″ W); and (iii) a plant nursery (Vivero Antoniucci, 38° 1′ 42.014″ S, 57° 37′ 59.374″ W). The area with the highest human exploitation and impact was Santa Paula’s Farms (SP), where 91.5% of the farm area was employed for intensive cultivation, mainly kiwis. The Natural Reserve Paititi (NRP) was characterized by the lowest human impact, considering that almost 20% of the area is a nature reserve characterized by abundant natural flora (with a large presence of *Eryngium regnelli*, *Baccharis tandilensis*, and *Lathyrus pubescebs*). Furthermore, 49% of the NRP is employed for extensive agriculture. The plant nursery Vivero Antoniucci (VA) showed a large amount of floral resources for pollinators, although the majority of them are cultivated plants not native to the site. Therefore, considering the level of anthropization, VA is at an intermediate stage between SP and NRP. Analyzing the total area taken into consideration for the study (3 km radius, corresponding to double the known flight radius of native bumble bees), it was observed that VA has 15% high-density residential areas, 4% urban reserves (biological corridors), and over 75% of intensive agricultural areas.

### Spatial Characterization

The spatial characterization of the three sampling areas was carried out using the Google Earth Engine platform and the open access software QGIS (https://qgis.org/). Landsat 8 satellite images were used with the atmospheric corrections made under the name “LANDSAT/LC08/C01/T1_TOA,” and only images with a cloudiness lower than 20% were considered. A representative image was constructed from the median of the images filtered in Google Earth Engine, which allowed working with a single image containing information from the entire sampling period. The constructed image was exported and processed with QGIS tools.

**Fig. 1:**
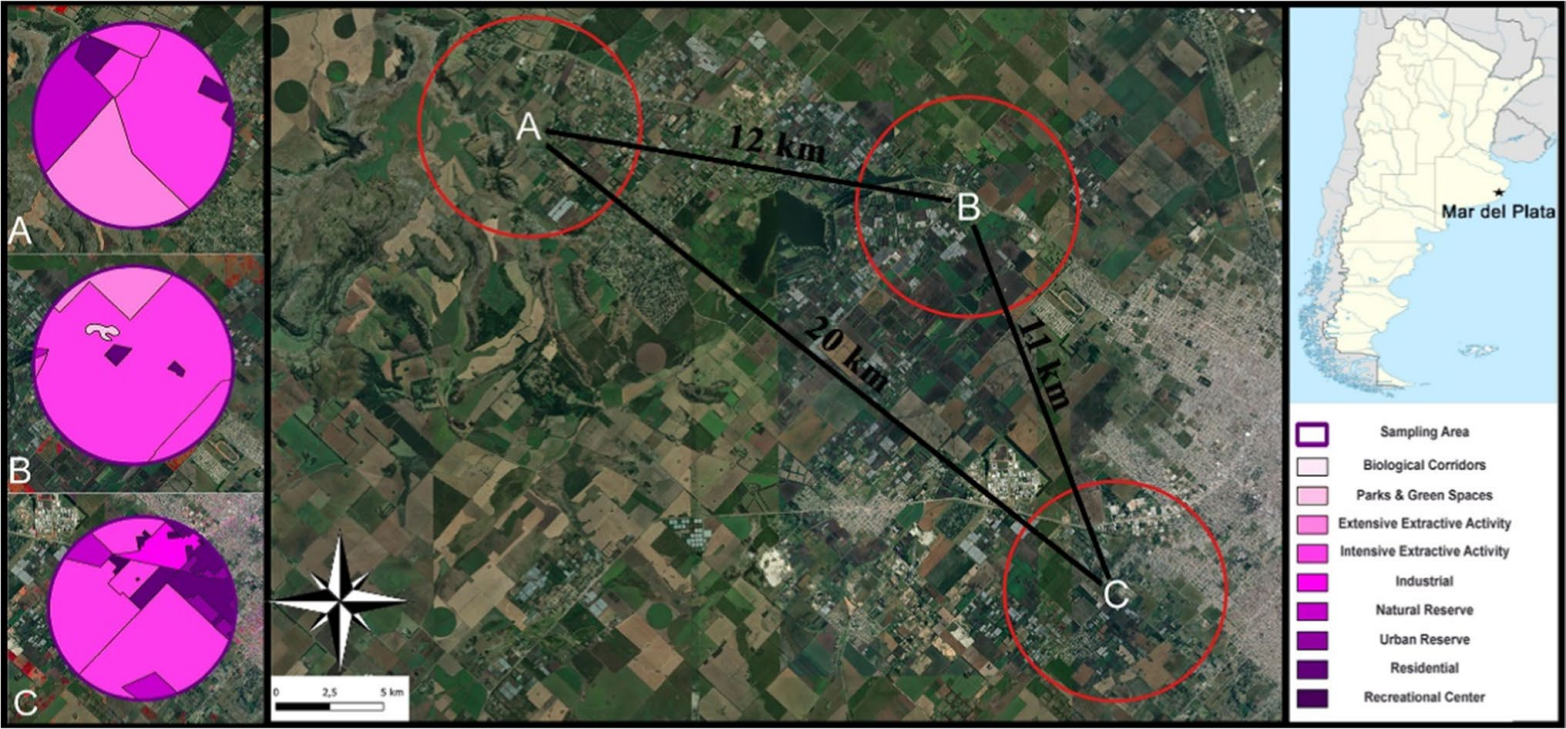
Spatial characterization. The central figure depicts the distribution of the three sampling sites, where A = Natural Reserve Paititi (NRP), B = Santa Paula’s Farm (SP), and C = Vivero Antoniucci (VA) (total distance between A-B and B-C > 10 km, and distance between A-C > 20 km). The three figures on the left side represent, in a yellow scale, the different land uses for each site (A-C) as declared in the national geographic information system database. On the right margin, color references are provided along with the corresponding land use types

Moreover, spatial characterization was based on land use information available in the Geographic Information System repository of the province of Buenos Aires “urbasig” (https://www.urbasig.gob.gba.gob.ar). This information consists of official reports from the government of the province of Buenos Aires, detailing the portions of territory used for different human activities. Three points corresponding to the three sampling zones were added to the final images in QGIS, from which a 3-km radius buffer was constructed according to the foraging area of the bumble bee species. Figure 1 summarizes the images detailed above.

### Bumble Bee Sampling

Bees were collected from December (2019) to March (2020); however, for downstream DNA sequencing, only bees sampled at the end of January were used. During this period, five collection campaigns were performed on the three chosen sites. During each sampling day, three transects of 70 m were delimited in each sampling site and bumble bees were collected in a time interval of 30 min per transect, during sunny days between 9 a.m. and 1 p.m. For this study, a total of ten female bumble bee workers per site were sampled directly from flowers through a homemade bee vacuum collector. Collected bees were placed individually in plastic vials at –20 °C until further analysis. Bee identification was carried out under a stereo microscope with 40 × magnification.

### DNA Extraction from Bumble Bee Gut, Pathogen Detection, and Real-Time PCR Analysis

Total genomic DNA was individually extracted from bumble bee guts using the High Pure PCR Template Preparation kit (Roche Diagnostics). The amount of extracted DNA is reported in Supplementary table S1. A PCR screening for bee pathogens *Nosema ceranae, Nosema apis, Nosema bombi, Crithidia bombi, Lotmaria passim, Apicystis bombi,* and *Apis mellifera* Filamentous Virus (AmFV) was carried out with specific primers reported in Supplementary Table 2, on all the extracted DNA samples. Only the detected pathogens were then quantified with StepOne™ Real-Time PCR System (Applied Biosystems) relying on the same primers reported in Supplementary Table 2, according to Fernandez de Landa et al. [27] and [40], with a minor modification: PowerUp™ SYBR™ Green Master Mix (Applied Biosystems, Life Technologies) was used as universal master mix, avoiding the initial step of Uracil-DNA glycosylases activation at 50 °C. DNA samples were diluted 1:10 prior to loading in the reaction plate. qPCR was used also to determine the total amount of bacteria (Eubacteria), according to [12]. The target amplicons melting temperature, annealing temperature and amplicon size are reported in Supplementary table S2, along with the primer pair sequences.

### NGS Analysis for the Gut Microbiome and Bioinformatics

The microbiome analysis on sampled bumble bees was performed with an NGS approach on an Illumina MiSeq sequencer and was based on the 16S *rRNA* gene, regions V5–V7. Libraries were prepared according to Alberoni et al. [8] with some modifications: briefly, KAPA Hi-Fi PCR Master Mix (Roche diagnostics) was used to amplify the target DNA with a maximum of 25 PCR cycles. The primers used are reported in Supplementary table S2 and allow the discrimination of the 16S rRNA gene amplicons of gut bacteria (about 470 bp) from amplicons deriving from pollen plastids (about 720 bp) according to Hanshew et al. [38]. The obtained PCR products were purified using magnetic beads (AMPure kit, Beckman Coulter) and E-Gel Size Select II 2% gel (Cat. Number G661012, ThermoFisher, Milan, Italy) according to Fernandez de Landa et al. [27].

Raw reads were analyzed with Qiime2 [15]. DADA2 [19] plugin was used for reads joining, denoising, and chimera check. With qiime feature-table filter-samples, samples with less than 20,000 reads had been removed. Reference reads were assigned using qiime feature-classifier classify-sklearn using full-length sequence classifier of Silva138 Database [57]. Bar plot and microbiome data at different taxonomic levels were obtained using qiime taxa barplot plugin. The tree was generated using qiime phylogeny align-to-tree-mafft-fasttree to use the rooted tree for alpha and beta diversity analysis. qiime diversity alpha-rarefaction script was used to obtain rarefaction curves. 

### Statistics

Linear models (LMs) have been used to compare the pathogens loads within species and the different sites. Since *B. bellicosus* was present only in the Paititi natural reserve, this bumble bee was excluded from the analysis. A model was fitted in which the response variables were the counts *N. ceranae*. The sample site was included as an explanatory variable to compare the effect of land use on gut microbiota composition of *B. pauloensis*; univariate and multivariate analyses were used. To perform univariate analysis, each genus of the gut microbiota, with at least 1% relative abundance, was tested for normality (Shapiro–Wilk test) and homogeneity of variance (Levene test). Depending on the assumptions of each genus, univariate comparisons were performed using ANOVA or Kruskal–Wallis followed by the Tukey test or Dunn test, respectively. Permutational multivariate analyses of variance (PERMANOVA) were performed based on the Bray–Curtis dissimilarity index applied to fourth-root transformed data (to reduce the weight of the most abundant genus) and 9999 permutations. All variables included in the PERMANOVA analysis were assessed for homogeneity assumption using the betadisper function from the vegan package [10]. A nonmetric multidimensional scaling (nMDS) based on Bray–Curtis distances and 20 minimum and 200 maximum random starts were also performed to corroborate the obtained results in PERMANOVA analysis [8]. These analyses were performed using “vegan” R package version 4.2.1. To analyze the relation between the pathogen *N. ceranae* and the gut microbiota, a Spearman correlation between the pathogen and each microbial genus encountered in the microbiome was calculated. Finally, PCA analysis was performed using packages FactoMineR and factoextra, taking into consideration 21 taxa at the species and/or genus level. All tests were two-tailed with a significance level of *p* ≤ 0.05. 

The statistical analysis of alpha and beta diversity NGS data was carried out with QIIME2 using vectors and matrixes of script “qiime diversity core-metrics-phylogenetic.” Alpha-index data were analyzed using R to check normality and variance distribution. Faith_PD and observed_features indexes being not normally distributed were analyzed using “qiime diversity alpha-group-significance” plugin. Evenessindex, with a normal distribution of data, was analyzed using R software with ANOVA analysis and posthoc test using lsmeans function. For beta diversity analysis, “Qiime diversity beta-phylogenetic” and “qiime diversity beta-group-significance” plugins were used (considering Generalized Unifrac and Bray–Curtis dissimilarity indexes).

The *p*-value considered for statistical analysis was adjusted using Bonferroni correction for three comparisons when comparing sampling sites (reference *p*-value 0.0167) and for six comparisons when comparing the four different groups of bumble bees collected in the different sites (reference *p*-value 0.0083). The *p*-values listed in the “Results” section are already corrected. Moreover, the degree of freedom (df) was calculated according to parametric and non-parametric statistics. On the contrary, with parametric statistics, the degree of freedom was calculated as the total number of samples, − 1 per experimental group.

DAA analysis to compare *B. bellicosus* and *B. pauloensis* in the NRP site was performed by filtering the otu table and using the plugins qiime composition ancombc and qiime composition da-barplot.

## Results

For the differential abundance analysis of *Bombus bellicosus* vs *Bombus pauloensis*, a total of 346 individuals of wild social bees were collected in this study. Among them, two *Bombus* species were identified: *Bombus pauloensis* (*n* = 323) and *Bombus bellicosus* (*n* = 23). While *Bombus pauloensis* was widely distributed across all study sites, allowing the collection of at least ten samples per site (analyzed bees, *n* = 30), *B. bellicosus* was only observed and sampled in the NRP site (analyzed bees, *n* = 23). Comparisons regarding bumble bee species and parasite load were performed only for individuals sampled at NRP since it was the only location where *B. bellicosus* were sampled. On the other hand, comparisons for the sites and the parasite load were performed only for *B. pauloensis* samples, since it was the only species present in all the study sites.

### The Identified Pathogens in the Analyzed Bumble Bees and Their Spatial Distribution in the Sampling Sites

**Fig. 2:**
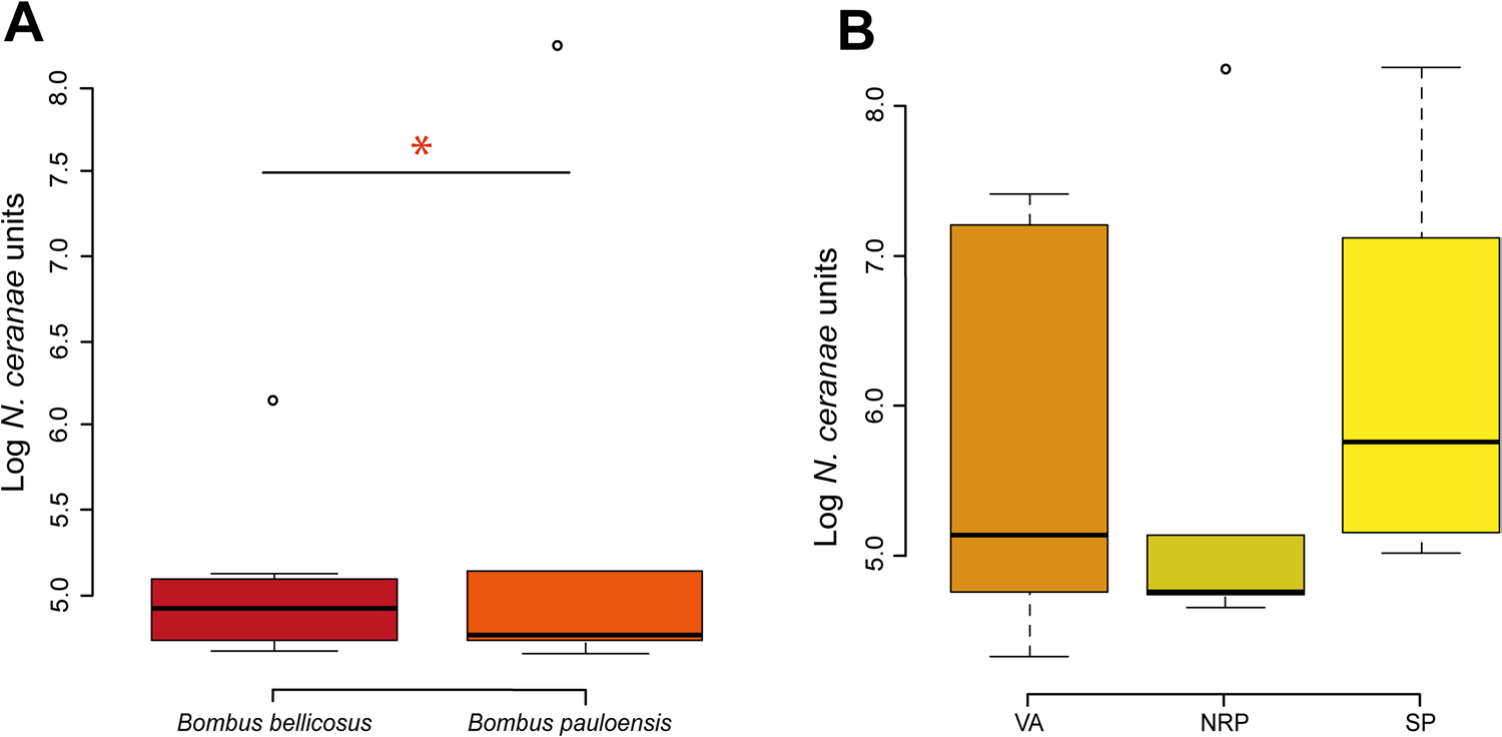
Box plot reporting the intensity of *N. ceranae* related to host species and sampling sites. **A** Comparison of *N. ceranae* units (NcU) with respect to the *B. bellicosus* (*n* = 10) and *B. pauloensis* (*n* = 10) sampled in NRP or **B** the environment. (*) Indicate statistically significant comparisons (*p* < 0.05, df: 30)

All the collected wild bumble bees were uninfected by *A. bombi*, *Ascosphaera* spp., *L. passim*, *N. apis*, and *AmFV* when analyzed in PCR. However, the samples resulted positive for *N. bombi* and *C. bombi* but the amplicon melting temperature did not correspond with the expeted one, therefore the species identification can not be considered reliable. Quantitative PCR showed an *N. ceranae* prevalence of 82.5% (results are available in Supplementary Table S1). The *N. ceranae* units (NcU) were statistically different within the two species of *Bombus*: Log 4.76 (± 1.23) and 4.02 (± 0.50) *N. ceranae* units (NcU) for *B. pauloensis* and *B. bellicosus*, respectively (ANOVA, *p* < 0.05, df: 1, Fig. 2A)*.* No statistically significant differences were highlighted comparing the different sites (Fig. 2B).

### Alfa and Beta Diversity Analysis

A total of 3,627,070 paired sequences were obtained from next-generation sequencing (NGS). Out of 40 sequenced samples, 32 had enough sequences allowing the downstream bioinformatic analysis: 8 samples of *B. bellicosus* and 24 samples of *B. pauloensis*. The obtained ASVs allowed a taxonomical assignation of bacterial taxa up to the genus level. The NGS results obtained for the analyzed bumble bees are summarized in Supplementary Table S3 at the phylum level, Supplementary Table S4 at the family level, and Supplementary Table S5 at the genus level. Only those bacterial genera with a relative abundance higher than 1% were considered for the analysis (according to [8] and [27]). Thus, the downstream analysis focused on 22 bacterial genera, while the less abundant taxa were grouped and categorized as “others.”

**Fig. 3:**
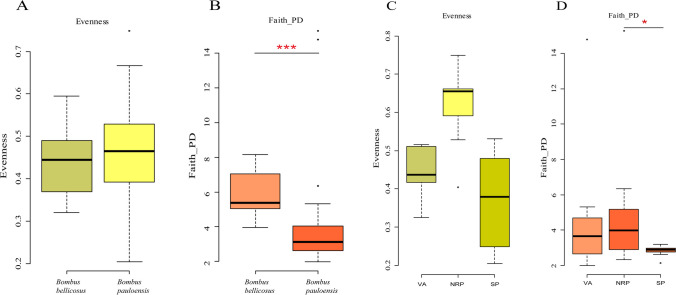
α-Diversity indexes. Comparing *B. pauloensis* of the three sampling sites (*n* = 24) and *B. bellicosus* of NRP (*n* = 8) with **A** Eveness and **B** Faith_PD. Comparing the different sampling sites Vivero Antoniucci (VA) vs Reserva Natural Paititi (NRP) vs Santa Paula (SP) with **C** Eveness and **D** Faith_PD. *p*-values: non-parametric tests, degrees of freedom typically relate to the number of data points involved in the analysis. For instance, in the Wilcoxon signed-rank test, which assesses differences between paired samples, the degrees of freedom are determined by the number of pairs minus one; *p < 0.05, ***p < 0.01

The alpha diversity analysis with Faith_PD, Eveness, and Observed_Features indexes underlined significant differences among bumble bee species and sampling sites (Fig. 3A–D; Fig. S2A-B).

Considering the two *Bombus* species in NRP, only the Evenness index showed a significant difference (*p* < 0.05, df: 1). Also comparing *B. pauloensis* in the three sampling sites, only the Evenness index turned out to be significant (*p* < 0.01, df: 2, Fig. 3C). Since the difference between the sampling sites for *B. pauloensis* is not significant for Faith_PD and Observed Features, for these indexes, we can take into consideration the entire dataset (*B. pauloensis* and *B. bellicosus*) to compare the two species. Considering the entire dataset, the two species differ in the phylogenetic composition of the microbiota (Faith-PD, *p* < 0.01; df: 1, Fig. 3B) and for the number of observed features (*p* < 0.01; df: 1, Fig. S2A).

The beta diversity analysis with Generalized Unifrac and Bray–Curtis dissimilarity indexes gave also significant results. PERMANOVA analysis on the Bray–Curtis dissimilarity index resulted in significant results considering both species and sites (Fig. S3A-S3B). When the sampling sites are considered, the pairwise comparison indicated that SP was significantly different *vs* NRP and VA (*p* < 0.05, df: 1). Considering the four different groups of bumble bees analyzed (*B. pauloensis* in three different sites and *B. bellicosus* in one site), the Bray–Curtis dissimilarity index evidenced a significant divergence comparing BellicosusNRP *vs* PauloensisSP groups (*p* < 0.01, df: 1), underlining a compositional dissimilarity between the two microbial communities. Moreover, Generalized Unifrac analysis shows a significant difference comparing PauloensisSP to PauloensisNRP and PaulonesisVA (*p* < 0.05, df: 1), suggesting an impact of the anthropic environment on the bumble bee microbiome) (Fig. S4A-D, and Supplementary Table S6).

### *B. pauloensis* Gut Microbiome

**Fig. 4:**
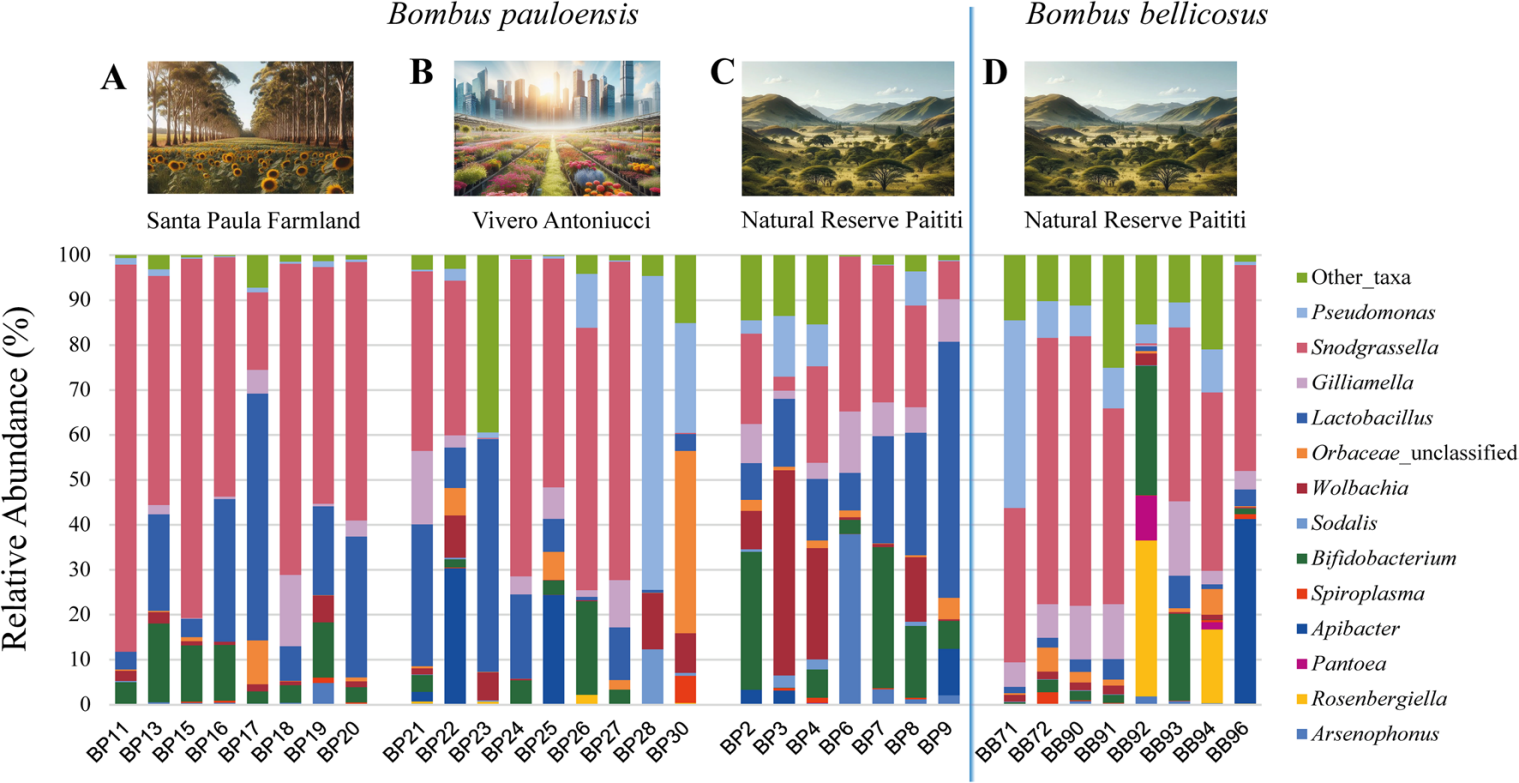
Relative abundance of the gut microbiota of *B. pauloensis* and *B. bellicosus* at a genus level. (A) Microbiome profiles for *B. pauloensis* in SP; (B) microbiome profiles for *B. pauloensis* in VA; (C) microbiome profiles for *B. pauloensis* in NRP; and (D) microbiome profiles for *B. bellicosus* in NRP

NGS sequences showed that the gut microbiome of *B. pauloensis* is mainly composed of four different phyla, namely Pseudomonadota (relative abundance 69.83%), Bacillota (21.38%), Actinomycetota (5.17%), and Bacteroidota (3.47%) (Fig. S5A). At the family level, Neisseriaceae (42.31%) and Lactobacillaceae (20.86%) resulted the most abundant, followed by Pseudomonadaceae (7.31%), Orbaceae (7.00%), Bifidobacteriaceae (4.84%), Weeksellaceae (3.43%), Morganellaceae (3.21%), Enterobacteriaceae (2.66%), Anaplasmataceae (1.82%), and Pectobacteriaceae (1.27%) (Fig. S5B). Finally, at the genus level, the gut microbiome of *B. pauloensis* is mainly composed of bacteria of the genera *Snodgrassella* (42.30%) and *Lactobacillus* (20.86%). With a lower relative abundance, the genera *Bifidobacterium* (4.82%), *Pseudomonas* (7.13%), *Wolbachia* (1.82%), and *Gilliamella* (3.32%) were detected (Fig. 4A–C). *Apibacter* (3.34%) and *Arsenophonus* (3.21%) were also present, although their prevalence in *B. pauloensis* was sporadic (7 out of 32 individuals for both *Apibacter* and *Arsenophonus*).

### *B. bellicosus* Gut Microbiome

The bacterial community of the sampled individuals of *B. bellicosus* was composed of the same four phyla detected in *B. pauloensis*, although their relative abundance was different: Pseudomonadota were the most abundant (83.49%), followed by Actinomycetota (5.88%), Bacteroidota (5.4%), and Bacillota (4.22%) (Fig. S6A). At the family level, as observed in the microbiota of *B. pauloensis*, the family with the highest relative abundance was Neisseriaceae (45.90%), followed by Pseudomonadaceae (14.70%), Erwiniaceae (8.33%), Orbaceae (7.81%), Weeksellaceae (5.38%), Bifidobacteriaceae (4.91%), Lactobacillaceae (3.57%), Morganellaceae (1.02%), and Rhodocyclaceae (1.05%) (Fig. S6B). At the genus level, *Snodgrassella* was the most abundant (45.88%), but unlike *B. pauloensis*, *Pseudomonas* (14.70%) was the second most abundant genus, followed by the genera *Gilliamella* (5.35%), *Bifidobacterium* (4.88%), *Rosenbergiella* (2.29%), *Apibacter* (5.34%), *Lactobacillus* (3.56%), *Pantoea* (3.77%), and *Wolbachia* (0.43%) (Fig. 4D). Although *Lactobacillus* presented low relative abundance values, it was the only bacterial genus found in 100% of the analyzed samples of *B. bellicosus*. On the other hand, *Snodgrassella*, *Gilliamella*, and *Pseudomonas* were detected in 88.90% of the samples analyzed.

### Comparison of Microbiomes Between *B. pauloensis*, *B. bellicosus*, and Land Use

The comparison among *B. pauloensis* and *B. bellicosus* bacterial genera showed significant differences in *Lactobacillus* that decreased from 20.68% in *B. pauloensis* to 3.56% in *B. bellicosus* (*p* < 0.05, df: 1), respectively. Interestingly, genera *Arsenophonus* and *Sodalis* showed a higher prevalence in *B. pauloensis* while they were completely absent from *B. bellicosus* (*p* < 0.05, df: 1). ASVs of the genus *Kluyvera* were detected only in one *B. pauloensis* (sample BP23 with 19.27% of relative abundance). On the contrary, the genera *Rosenbergiella* and *Pantoea* were detected only in *B. bellicosus* (*p* < 0.05, df: 1) with 2.29% and 3.77% of relative abundance, respectively. Therefore, these two genera (*Rosenbergiella* and *Pantoea*) are suggested as specific core members of *B. bellicosus* gut microbiome.

The comparison of the gut microbiome composition of *B. pauloensis* sampled in the different sites showed significant differences only for minor genera *Brevudimonas*, *Sodalis*, and *Methyloversatilis* when NRP is compared to SP and VA (*p* < 0.05, df: 1). All the other detected genera showed no statistical difference between the study sites. The DAA analysis (Fig. S7) considering *B. bellicosus* and *B. pauloensis* in natural environment (NRP) revealed a depletion of 2 LFC (Log fold change) for the genus *Arsenophonus* in *B. bellicosus* and an increase of more than 3 LFC for the genera *Microbacterium*, *Geothermobacter*, *Geobacter*, *Methyloversatilis*, *Comamonadaceae*, *Clostridium*, *Propinivibrio*, and *Dechloromonas*.

**Fig. 5:**
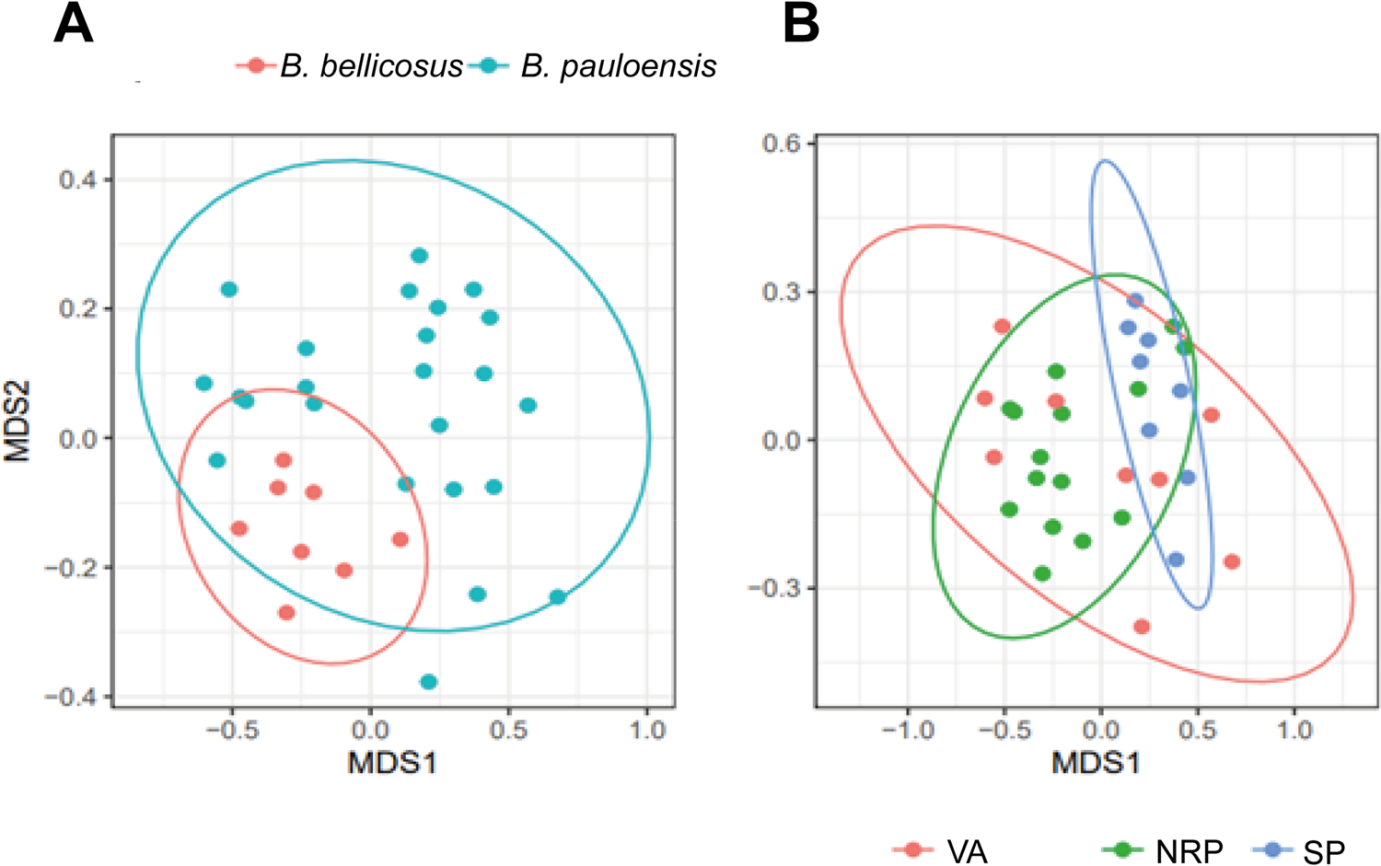
Variation in the gut microbiota assemblage of *B. pauloensis* and *B. bellicosus*. Results of the PERMANOVA analysis for the variation of the gut bacterial assemblage with respect to the **A** different species studied and **B** the different land uses

Nonmetric multidimensional scaling (NMDS) showed a nonmetric fit of *r*^2^ = 0.978, as the confidence level on the iteration stress test. The microbiome of both wild bumble bees shows statistically significant differences (PERMANOVA, *F* = 2.37, *r*^2^ = 0.07, and *p* < 0.05, df: 1) (Fig. 5A). However, the same NMDS analysis demonstrated that the intestinal microbial assemblage of bumble bees did not vary significantly in the different sites (PERMANOVA, *F* = 1.02, *r*^2^ = 0.23, and *p* > 0.05, df: 1, Fig. 5B) as shown by the overlapping of the confidence ellipses corresponding to the study sites. This finding suggests that the different land uses does not significantly influence the overall composition of the bumble bee intestinal microbiome taking into consideration the two studied species grouped (*B. pauloensis* of NRP–SP–VA compared to *B. bellicosus* of NRP).

### The Gut Pathogens Contribute to the Shaping of the Gut Microbiome of *B. pauloensis*

**Fig. 6:**
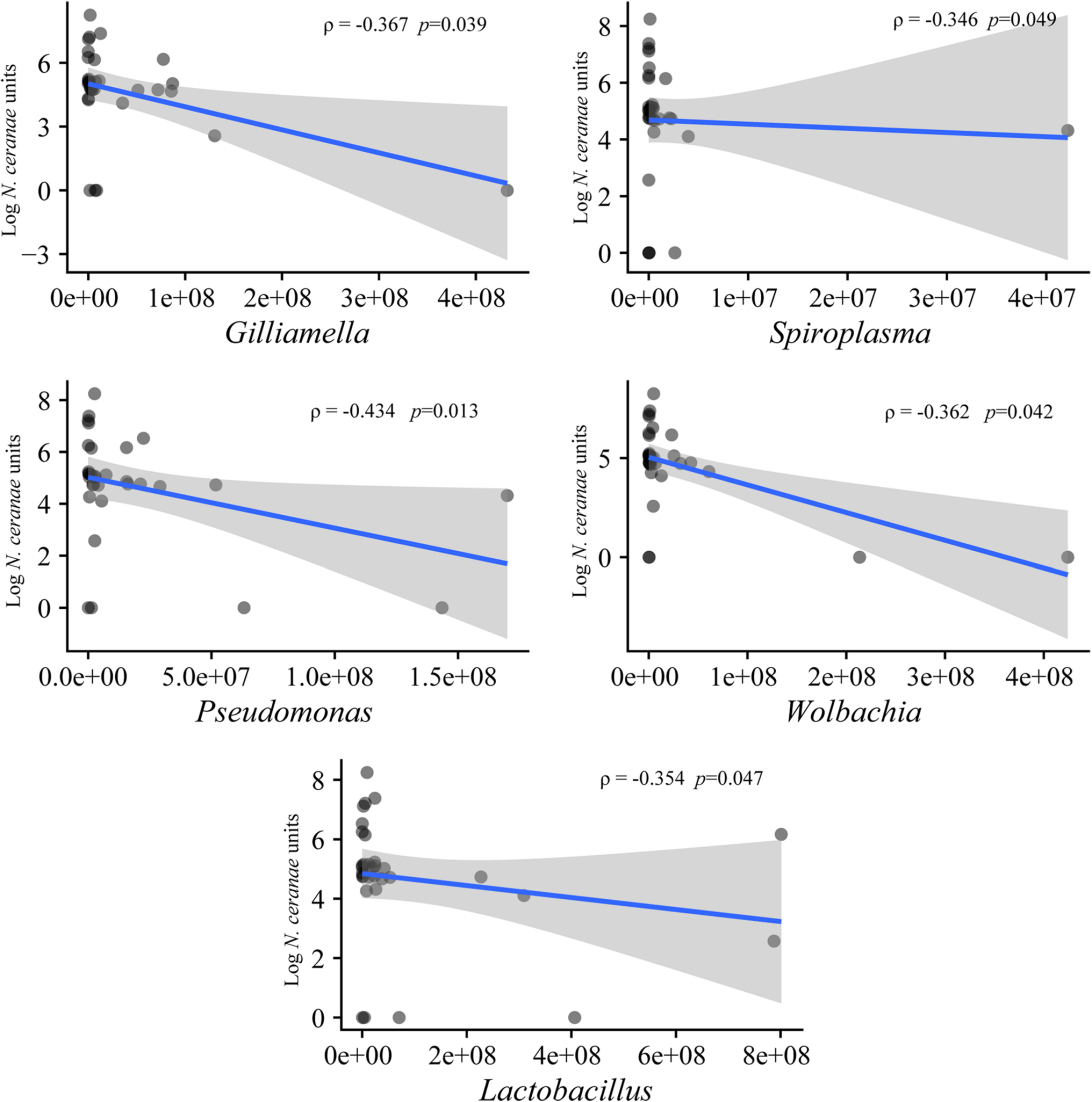
Pathogens and intestinal bacteria correlation. The dot plot shows the correlations between the *N. ceranae* units (expressed as the logarithm of the absolute quantification of both spores and vegetative forms) and the relative abundances of the significant bacterial genera studied that showed statistical significance (*p* ≤ 0.05)

The microbial taxa detected in the *B. pauloensis* gut revealed different interactions and correlation confidence levels with *N. ceranae*, reporting negative significant correlations between *Gilliamella* (Spearman’s correlation value =  − 0.33), *Spiroplasma* (Spearman’s correlation value =  − 0.35), *Pseudomonas* (Spearman’s correlation value =  − 0.43), *Wolbachia* (Spearman’s correlation value =  − 0.36), and *Lactobacillus* (Spearman’s correlation value =  − 0.37) (Fig. 6). At the same time, genera *Spiroplasma* and *Rosenbergiella* showed a negative correlation with *N. bombi* (Spearman’s correlation values =  − 0.68 for both genera). Conversely, *N. bombi* is positively correlated with the genus *Methyloversatilis* (Spearman’s correlation values = 0.36), while negative correlations were highlighted with the genera *Rosenbergiella* and *Spiroplasma* (*p* > 0.05, df: 1). Like *Methyloversatilis*, the genus *Apibacter* exhibited positive and significant correlation with a parasite, in this case *C. bombi* (Spearman’s correlation value =  − 0.36). Differently to previous studies [48, 56], other gut microbial taxa did not show any significant correlation with *C. bombi*.

## Discussion

The present work focuses on the structural composition of the microbiome of two bumble bee species (*B. pauloensis* and *B. bellicosus* (Fig. S7 and Fig. S8)) widely distributed in South America. This work was carried out in the Pampas region, where a strong anthropic pressure affected the entire ecosystem, destroying the nesting sites and trophic resources available for wild pollinators. Recent works showed how the microbiome of soil, plants, and pollinators are strictly interconnected [51] and dependent on environmental stressors and human activities [13, 26]. Therefore, our research work aimed at correlating the native bumble bee’s microbiome with the land use and gut pathogens load that may lead to dysbiosis [6, 50]. Of particular relevance is the focus on the gut microbiome of *B. pauloensis,* a bumble bee relevant for commercial pollination purposes. This species is considered the natural and environment-adapted substitute of the European bumble bee species *Bombus terrestris* (exogenous of South America), and its boosting is necessary to preserve the neotropical region from exogenous invaders. Moreover, our study describes for the first time the gut microbiome composition of *B.* *bellicosus*, a vulnerable species [46] and extinct in some South American regions such as Paranà (The IUCN Red List of Threatened Species).

Previous investigations on bumble bee microbiome were mainly focused on European and North American bumble bee species, such as *Bombus bimaculatus*, *B. griseocollis*, *Bombus impatiens* [21], and *B. terrestris* [16]. These bumble bee species showed a microbiome usually composed of five core microbial taxa: *Bifidobacterium*, *Gilliamella, Lactobacillus, Schmidhempelia*, and *Snodgrassella* [14, 35–37]. The gut microbiome of *B. pauloensis* and *B.* *bellicosus* is similar to the microbiome described in other bumble bee species*.* However*, B. pauloensis* and *B.* *bellicosus* microbiomes showed differences when compared with each other, in both abundance and diversity of core microbial taxa. The most abundant genus in both species was *Snodgrassella* which had a relative abundance higher than 40%. The second predominant taxon was *Lactobacillus* in *B. pauloensis*, whereas *Pseudomonas* was the second predominant in *B. bellicosus.* In honeybees, lactobacilli (Firm 4 and Firm 5) are known to produce short-chain fatty acids [73] useful for pollinators as a source of energy, behavioral modulation [70], and chitinases [9]. These lactobacilli are likely closely related to the lactobacilli found in the analyzed bumble bees. However, the low *Lactobacillus* abundance in *B.* *bellicosus* was unexpected*.* This microbial taxon was probably replaced by *Pseudomonas,* a controversial opportunistic taxon when detected in the insect gut microbiome [23]*.* The genus *Pseudomonas* was recently found to populate the gut microbiome of solitary bees in the same Neotropical region [27, 41], in honeybees populating semi-desertic environment [30], or in bees treated with antibiotics [12]. However, this taxon in bumble bees was recently related to microbiome disruption [66] and therefore to compromised bee health [6]. Since *B. bellicosus* is an endangered species, our finding seems to confirm the high susceptibility of this pollinator to adverse environmental factors.

Another indicator of the health status of the sampled *B. bellicosus* is represented by the relative abundance of opportunistic environmental bacteria (here defined as “Other_taxa”), or non-core members which are highly abundant in this bumble bee species (above 10% in *B. bellicosus vs* below 4% in *B. pauloensis*). Among the opportunistic bacteria, the order Enterobacteriales was found to be the most abundant. Along with Pseudomonadaceae, also Enterobacteriaceae and Yersiniaceae have been previously correlated with environmental stressors [59], disrupted microbiome [66], or septicemia [17]. Our analysis detected Enterobacteriaceae in nine individuals, seven of them belonging to the NRP environment and two to the VA, often co-occurring with *Pseudomonas*. Among opportunistic taxa, Erwiniaceae (*Pantoea* and *ZDC*) were preferably populating *B. bellicosus*, while *Sodalis* (Pectobacteriaceae) showed a higher prevalence in *B. pauloensis*. *Pantoea* was recently described as a low-occurring taxa in *B. terrestris*, and its presence seems to be shaped by the local trophic resources [68]. However, currently, there is a knowledge gap on the role of *Rosenbergiella* and *Sodalis* in the bumble bee gut. The incidence of these environmental bacteria is probably indicating interactions between these bumble bees with other pollinators through flowers [42].

Comparing the gut microbiome of *B. pauloensis* and *B. bellicosus* with the microbial profile of other American bumble bee species such as *Bombus hortulanus* and *Bombus rubicundus* [66], the only difference detected was the absence of the genus *Schmidhempelia*. However, it is known that the relative abundance of *Schmidhempelia* drastically decreases during aging [36, 37]. Considering that bumble bees sampled in our study were all foragers, the absence of *Schmidhempelia* might be associated with aging and therefore coherent with previous findings [36, 37, 68]. The differences between the gut microbiomes of *B. pauloensis* and *B. bellicosus* might be influenced by pathogen load. *N. ceranae* was present with a high prevalence and intensity in the analyzed bumble bees, as already described by Arbulo et al. [11] and Plischuk et al. [55]. Authors such as Ngor et al. [49] and Gisder et al. [33] have demonstrated that even when exposed to doses previously considered optimal for the development of parasitosis (from 6500 to above 100,000 spores), individuals of different bumble bee species from the northern hemisphere (*Bombus terrestris* and *Bombus impatiens*) did not exhibit symptoms of infection. Therefore, the detection of DNA in the individuals collected in this study would not be sufficient to indicate an impacting or lethal infection.

In our study, the correlation between the studied pathogen and the gut microbiota of *B. pauloensis* and *B. bellicosus* was attempted. The abundance of the genus *Gilliamella* was found to be negatively correlated with *N. ceranae*, in contrast with the findings described by Rubanov et al. [60] and Sbaghdi et al. [61], who identified various strains of *Gilliamella* that were positively correlated with high infections of *N. ceranae*. However, our findings are consistent with previous studies of Kwong et al. [45] and Zheng et al. [72], which proposed the gut acidification by *Gilliamella* as a barrier against gut pathogens. Studies correlating the genus *Gilliamella* with various pathogens are recent, and clear trends or precise explanations for these correlations are not yet established. Therefore, future research is necessary to precisely elucidate the role of *Gilliamella* in the gut of pollinators and its interaction with parasites. This understanding is crucial for developing comprehensive strategies to support current methodologies aimed at enhancing the immune systems of both honeybees and non-*Apis* pollinators. Regarding the interaction between *N. ceranae* and the genus *Lactobacillus*, recent literature supports the results evidenced here. On one hand, Tejerina et al. [63] observed that the supplementation of *A. mellifera* colonies located in northern Argentina with preparations of the species *Lactobacillus salivarius* contributed to the control of both *N. ceranae* and the ectoparasite *Varroa destructor*. On the other hand, Yu et al. [69] demonstrated how the supplementation of *B. terrestris* individuals with *Lactobacillus melliventris* promoted improved immune systems and reduced parasitosis rates. However, the results obtained here do not explain causality but rather correlation. Future studies should be conducted to investigate and delve deeper into the interaction between species of the genus *Lactobacillus* and a widely distributed pathogenic agent such as *N. ceranae.* Finally, to the best of our knowledge, this research represents the first evidence of a negative correlation between *Spiroplasma, Wolbachia*, and *Pseudomonas* and the parasitic intensities of *N. ceranae*. As mentioned previously, the interactions between the bacterial taxa and the microsporidian *N. ceranae* discussed here do not demonstrate a causal relationship. Therefore, the development of future studies to determine whether these bacterial groups can indeed contribute to the regulation of Nosemosis development in *Bombus* bees is of great interest. This could lead to the development of new, comprehensive, and holistic strategies for parasite control using biological-based technologies.

Our study provides a first insight into the structural composition of the core microbiome of two widely distributed bumble bee species in South America, *B. pauloensis* and *B. bellicosus*. The characterization of the gut microbiome of *B. bellicosus*, an endangered species, raises the importance of profiling the core gut microbial community and the understanding of microbial dynamics involved in a threatened species. Alpha and beta diversity indexes supported the hypothesis of the possible influence of anthropized environments being the species and site variables significant when comparing *B. bellicosus vs B. pauloensis* and *B. pauloensis* in NRP vs *B. pauloensis* in SP. Bumble bees collected from each sampling site necessarily belong to different colonies due to the distances between the sites (greater than the foraging radius of bumble bees). However, this factor has a minor impact if compared with anthropogenic factors on the changes and variability within bacterial communities. In fact, the microbiome of social corbiculate bees tends to remain constant between populations, despite geographical distance [5, 44]. A limitation of this research is represented by a lack of replicates considering sampling sites (especially anthropized ones). Therefore, future research efforts should focus on elucidating the intricate interactions between bumble bee microbiota, pathogens, and environmental factors at a higher scale, differentiating land use, the effect of distance, and dispersal limitation of microorganisms.

## Supplementary Information

Below is the link to the electronic supplementary material.

Supplementary file1 (PDF 1.03 MB) 

## Data Availability

NGS raw sequence data have been submitted to NCBI repository under the Sequence Read Archive (SRA) databases under the Bioproject N° PRJNA799463. Accession numbers SRR17715401, SRR17715406—SRR17715434, SRR17715437, SRR17715448, SRR17715459, SRR17715470, SRR17715481 and SRR17715482. Please refer to Supplementary Table S7 to identify the samples accession number.
