# Supplementary material for: The Gut Microbiome of Two Wild Bumble Bee Species Native of South America: Bombus pauloensis and Bombus bellicosus
Source: Microb Ecol. 2024 Sep 28;87:121. doi: 10.1007/s00248-024-02430-y (PMC11438738; doi:10.1007/s00248-024-02430-y)
Supplement: Supplementary file 1 [file 248_2024_2430_MOESM1_ESM.pdf]

Supplementary materials for:

The gut microbiome of two wild bumblebee species native of South America: *Bombus pauloensis* and *Bombus bellicosus*

Gregorio Fernandez De Landa[1,2], Daniele Alberoni[3*], Chiara Braglia[3], Loredana Baffoni[3], Mateo Fernandez de Landa[1,2], Pablo Revainera[1,2], Silvina Quintana[1,2], Francisco Zumpano[4], Matias Maggi[1,2], Diana Di Gioia[3].

[1] Facultad de Ciencias Exactas y Naturales, Centro Científico Tecnológico Mar del Plata, CONICET, Centro de Asociación Simple CIC PBA,Instituto de Investigaciones en Producción Sanidad y Ambiente (IIPROSAM), Facultad de Ciencias Exactas y Naturales, Universidad Nacional de Mar del Plata, Centro Científico Tecnológico Mar del Plata, CONICET. Centro de Asociación Simple CIC PBA, Mar del Plata, Argentina.

[2] Centro de Investigaciones en Abejas Sociales, Facultad de Ciencias Exactas y Naturales, Universidad Nacional de Mar del Plata, Mar del Plata, Argentina.

[3] Dipartimento di Scienze e Tecnologie Agro-Alimentari, University of Bologna, Viale Fanin 44, 40127, Bologna, Italy.

[4] Instituto de Investigaciones Marinas y Costeras (IIMyC), Facultad de Ciencias Exactas y Naturales, Universidad Nacional de Mar del Plata-CONICET. Funes 3350 Mar del Plata (7600), Argentina

* Corresponding Author: daniele.alberoni@unibo.it

Supplementary table S1: The table describes qPCR results on the gut pathogen *N. ceranae* and Eubacteria expressed in Log, and the amount of DNA extracted per sample expressed in ng/µl.

Species	Sampling Site	DNA Quantification (ng/µl)	*Nosema ceranae* units (Log)	Eubacteria (Log)
B. pauloensis	NRP	5.78	0.00	7.385
B. pauloensis	NRP	1.84	0.00	7.613
B. pauloensis	NRP	3.19	0.00	8.520
B. pauloensis	NRP	12.40	4.89	8.449
B. pauloensis	NRP	72.00	0.00	9.726
B. pauloensis	NRP	11.60	0.00	9.299
B. pauloensis	NRP	75.3	4.82	8.187
B. pauloensis	NRP	48.7	8.24	10.207
B. pauloensis	NRP	7.67	5.14	9.576
B. pauloensis	NRP	47.50	4.89	8.436
B. pauloensis	SP	163.00	6.29	8.998
B. pauloensis	SP	30.80	5.26	8.260
B. pauloensis	SP	217.00	7.17	8.067
B. pauloensis	SP	8.87	4.35	9.446
B. pauloensis	SP	31.30	5.42	8.790
B. pauloensis	SP	86.70	5.26	8.600
B. pauloensis	SP	22.50	6.15	8.325
B. pauloensis	SP	15.80	5.17	9.138
B. pauloensis	SP	221.00	8.52	9.394
B. pauloensis	SP	15.7	8.21	8.743
B. pauloensis	VA	9.47	7.44	8.363
B. pauloensis	VA	7.13	5.21	9.490
B. pauloensis	VA	27.20	4.86	8.063
B. pauloensis	VA	27.10	7.19	9.713
B. pauloensis	VA	3.44	4.9	7.385
B. pauloensis	VA	3.00	4.55	7.613
B. pauloensis	VA	9.47	5.13	8.520
B. pauloensis	VA	2.01	6.59	8.449
B. pauloensis	VA	14.00	7.45	9.726
B. pauloensis	VA	109.00	4.25	9.299
B. belicosus	NRP	14.90	4.84	8.169
B. belicosus	NRP	3.03	4.47	7.727
B. belicosus	NRP	1.68	5.01	8.064
B. belicosus	NRP	1.88	4.67	7.689
B. belicosus	NRP	1.15	0.00	9.507
B. belicosus	NRP	14.30	4.72	9.213
B. belicosus	NRP	15.00	4.93	7.974
B. belicosus	NRP	1.35	4.87	9.792
B. belicosus	NRP	10.30	0.00	8.169
B. belicosus	NRP	15.20	6.06	7.727

Supplementary table S2: Table with complete list of primers used for PCR and qPCR analysis

TARGET	PRIMER NAME	SEQUENCE	AMPLICON MELTING TEMPERATURE	ANNEALING TEMPERATURE	AMPLICON SIZE	References
Lotmaria passim	qCrFw1 153	5'- TCCACTCTGCAAACGATGAC -3'	87.0	56	153	Arismendi et al., 2016
	qCrRev1 153	5'- GGGCCGAATGGAAAAGATAC -3'				
Ascosphaera spp.	AscoAll1	5'- GCACTCCCACCCTTGTCTA -3'	89.8	58	550	James & Skinner 2005
	AscoAll2	5'- GAACACGACGCCGTCACT -3'				
Acarapis woody	AcarFor	5'- CGGGCCCGAGCTTATTTTACTGCTG -3'	78.5	57	162	Garrido-Bailónet al., 2012
	AcarRev	5'- GCGCCTGTCAATCCACCTACAGAA -3'				
Nosema apis	321 apis Fw	5'- GGGGGCATGTCTTTGACGTACTATGTA -3'	84.3	60	321	Martín-Hernández et al., 2007
	321 apis Rev	5'- GGGGGGCGTTTAAAATGTGAAACAACTATG -3'				
Nosema cerenae	218mitoc Fw	5'- CGGCGACGATGTGATATGAAAATATTAA -3'	77.0	60	218	Martín-Hernández et al., 2007
	218mitoc Rev	5'- CCCGGTCATTCTCAAACAAAAAACCG -3'				
Apicystis bombi	ApBF1293 Fw	5'- CGTACTGCCCTGAATACTCCAG -3'	81.7	58	293	Meeus et al, 2018
	ApBR1293 Rv	5'- TGAAAGCGGCGTATACATGA -3'				
Crithidia bombi	Crithidia119fw	5'- CCAACGGTGAGCCGCATTCAGT -3'	83.7	58	119	Huang et al., 2015
	Crithidia119rv	5'- CGCGTGTCGCCCAGAACATTGA -3'				
Nosema bombi	NBom323 fw	5'- CCATGCATGTTTTTGAAGATTATTAT -3'	78.1	56	323	Klee et al., 2006
	NBom 323 rv	5'- CATATATTTTTAAAATATGAAACAATAA -3'				
AmFV	AmFV 551 -F	5'- CAGAGAATTCGGTTTTTGTGAGTG -3'	84.7	52	551	Hartmann et al., 2015
	AmFV 551 -R	5'- CATGGTGGCCAAGTCTTGCT -3'				
Eubacteria	Eub338-F	5'- ACTCCTACGGGAGGCAGCAG-3'	-	60	200	Favaro et al., 2023
	Eub518-R	5'- ATTACCGCGGCTGCTGG -3'				
16S rRNA region V7-V8 with adapters	799F	5'- TCGTCGGCAGCGTCAGATGTGTATAAGAGACAGAACMGGATTAGATACCCG -3			470 and 720	
	1139R	5'- GTCTCGTGGGCTCGGAGATGTGTATAAGAGACAGACGTCATCCCCACCTTC -3				

Supplementary table S3: Relative abundance at Phylum level. The sampling sites reported: Natural Reserve Paititi (NRP); Santa Paula´s Farm (SP); Vivero Antoniucci (VA)

**Sample**			**Phylum**				
**Code**	**Sampling site**	**Bumblebee Specie**	**Proteobacteria**	**Firmicutes**	**Bacteroidota**	**Actinobacteriota**	**Others**
BB71	NRP	*B. bellicosus*	0.9573	0.0208	0.0008	0.0082	0.0129
BB72	NRP	*B. bellicosus*	0.9346	0.0346	0.0017	0.0229	0.0063
BB90	NRP	*B. bellicosus*	0.9385	0.0336	0.0007	0.0173	0.0099
BB91	NRP	*B. bellicosus*	0.8773	0.0612	0.0016	0.0424	0.0176
BB92	NRP	*B. bellicosus*	0.7529	0.0164	0.0010	0.2250	0.0047
BB93	NRP	*B. bellicosus*	0.7626	0.0947	0.0001	0.1302	0.0124
BB94	NRP	*B. bellicosus*	0.9318	0.0347	0.0000	0.0179	0.0157
BB96	NRP	*B. bellicosus*	0.5242	0.0412	0.4267	0.0069	0.0010
BP02	NRP	*B. pauloensis*	0.6551	0.1022	0.0449	0.1955	0.0023
BP04	NRP	*B. pauloensis*	0.7568	0.1800	0.0038	0.0525	0.0069
BP06	NRP	*B. pauloensis*	0.9106	0.0751	0.0001	0.0143	0.0000
BP07	NRP	*B. pauloensis*	0.5060	0.2953	0.0018	0.1960	0.0009
BP08	NRP	*B. pauloensis*	0.5721	0.3259	0.0024	0.0988	0.0009
BP09	NRP	*B. pauloensis*	0.2460	0.6098	0.1105	0.0336	0.0001
BP11	SP	*B. pauloensis*	0.9217	0.0405	0.0004	0.0253	0.0120
BP13	SP	*B. pauloensis*	0.6517	0.2444	0.0000	0.1029	0.0010
BP15	SP	*B. pauloensis*	0.8879	0.0445	0.0003	0.0673	0.0000
BP16	SP	*B. pauloensis*	0.5903	0.3432	0.0000	0.0665	0.0000
BP17	SP	*B. pauloensis*	0.3713	0.6017	0.0025	0.0240	0.0005
BP18	SP	*B. pauloensis*	0.8913	0.0847	0.0006	0.0234	0.0000
BP19	SP	*B. pauloensis*	0.7091	0.2211	0.0004	0.0695	0.0000
BP20	VA	*B. pauloensis*	0.6511	0.3296	0.0000	0.0193	0.0000
BP21	VA	*B. pauloensis*	0.5903	0.3571	0.0258	0.0262	0.0007
BP22	VA	*B. pauloensis*	0.5556	0.0991	0.3313	0.0136	0.0004
BP23	VA	*B. pauloensis*	0.5103	0.4864	0.0003	0.0026	0.0004
BP24	VA	*B. pauloensis*	0.7719	0.1985	0.0002	0.0292	0.0004
BP25	VA	*B. pauloensis*	0.6510	0.0766	0.2555	0.0168	0.0000
BP26	VA	*B. pauloensis*	0.8704	0.0089	0.0000	0.1197	0.0010
BP27	VA	*B. pauloensis*	0.8560	0.1258	0.0000	0.0182	0.0000
BP28	VA	*B. pauloensis*	0.9910	0.0048	0.0010	0.0015	0.0016
BP03	NRP	*B. pauloensis*	0.7293	0.2142	0.0454	0.0086	0.0024
BP30	VA	*B. pauloensis*	0.9120	0.0617	0.0047	0.0148	0.0069

Supplementary table S4: Relative abundance at Family level. The sampling sites reported: Natural Reserve Paititi (NRP); Santa Paula´s Farm (SP); Vivero Antoniucci (VA)

Sample			Family										
Code	Sampling site	Bumblebee Specie	Neisseriaceae	Pseudomonadaceae	Morganellaceae	Enterobacterales	Lactobacillaceae	Orbaceae	Weeksellaceae	Bifidobacteriaceae	Anaplasmataceae	Pectobacteriaceae	Erwiniaceae
BB71	NRP	*B. bellicosus*	0.348	0.517	0.000	0.025	0.014	0.037	0.001	0.002	0.004	0.002	0.000
BB72	NRP	*B. bellicosus*	0.665	0.112	0.000	0.021	0.024	0.110	0.000	0.017	0.005	0.002	0.000
BB90	NRP	*B. bellicosus*	0.675	0.094	0.013	0.018	0.031	0.107	0.001	0.013	0.005	0.002	0.001
BB91	NRP	*B. bellicosus*	0.523	0.133	0.005	0.037	0.055	0.104	0.001	0.011	0.006	0.002	0.000
BB92	NRP	*B. bellicosus*	0.006	0.079	0.042	0.016	0.016	0.011	0.001	0.220	0.010	0.004	0.571
BB93	NRP	*B. bellicosus*	0.495	0.088	0.015	0.009	0.093	0.137	0.000	0.124	0.001	0.000	0.000
BB94	NRP	*B. bellicosus*	0.491	0.145	0.006	0.053	0.013	0.092	0.000	0.000	0.003	0.000	0.091
BB96	NRP	*B. bellicosus*	0.478	0.010	0.001	0.002	0.038	0.028	0.427	0.007	0.001	0.001	0.003
BP02	NRP	*B. pauloensis*	0.248	0.044	0.001	0.030	0.101	0.095	0.044	0.190	0.026	0.013	0.000
BP03	NRP	*B. pauloensis*	0.043	0.226	0.004	0.145	0.206	0.026	0.045	0.001	0.156	0.065	0.001
BP04	NRP	*B. pauloensis*	0.267	0.143	0.006	0.122	0.171	0.048	0.003	0.039	0.077	0.048	0.001
BP06	NRP	*B. pauloensis*	0.309	0.001	0.510	0.002	0.075	0.087	0.000	0.014	0.001	0.000	0.000
BP07	NRP	*B. pauloensis*	0.375	0.003	0.064	0.004	0.294	0.057	0.002	0.196	0.002	0.000	0.000
BP08	NRP	*B. pauloensis*	0.269	0.110	0.022	0.051	0.324	0.045	0.002	0.095	0.043	0.019	0.000
BP09	NRP	*B. pauloensis*	0.091	0.003	0.034	0.002	0.610	0.112	0.111	0.034	0.001	0.000	0.000
BP11	SP	*B. pauloensis*	0.889	0.019	0.001	0.010	0.041	0.003	0.000	0.026	0.006	0.004	0.000
BP13	SP	*B. pauloensis*	0.579	0.020	0.010	0.013	0.245	0.017	0.000	0.100	0.007	0.000	0.000
BP15	SP	*B. pauloensis*	0.860	0.004	0.006	0.001	0.044	0.011	0.000	0.067	0.003	0.000	0.001
BP16	SP	*B. pauloensis*	0.572	0.003	0.007	0.002	0.341	0.003	0.000	0.067	0.002	0.000	0.000
BP17	SP	*B. pauloensis*	0.189	0.014	0.004	0.009	0.603	0.142	0.002	0.015	0.004	0.000	0.000
BP18	SP	*B. pauloensis*	0.762	0.007	0.007	0.004	0.085	0.106	0.000	0.021	0.002	0.000	0.000
BP19	SP	*B. pauloensis*	0.581	0.018	0.080	0.008	0.218	0.005	0.000	0.068	0.016	0.000	0.000
BP20	VA	*B. pauloensis*	0.603	0.007	0.002	0.003	0.328	0.032	0.000	0.018	0.003	0.000	0.000
BP21	VA	*B. pauloensis*	0.451	0.005	0.005	0.004	0.357	0.116	0.026	0.021	0.004	0.002	0.001
BP22	VA	*B. pauloensis*	0.375	0.036	0.000	0.020	0.098	0.084	0.331	0.010	0.026	0.007	0.000
BP23	VA	*B. pauloensis*	0.001	0.012	0.002	0.012	0.417	0.002	0.000	0.000	0.012	0.004	0.001
BP24	VA	*B. pauloensis*	0.742	0.001	0.000	0.002	0.197	0.026	0.000	0.029	0.001	0.000	0.000
BP25	VA	*B. pauloensis*	0.533	0.007	0.000	0.000	0.076	0.109	0.256	0.017	0.000	0.000	0.000
BP26	VA	*B.pauloensis*	0.669	0.168	0.001	0.004	0.009	0.010	0.000	0.119	0.001	0.000	0.006
BP27	VA	*B. pauloensis*	0.759	0.004	0.000	0.000	0.126	0.090	0.000	0.018	0.000	0.000	0.000
BP28	VA	*B. pauloensis*	0.000	0.520	0.000	0.315	0.004	0.000	0.001	0.000	0.019	0.129	0.000
BP30	VA	*B. pauloensis*	0.003	0.339	0.003	0.047	0.042	0.459	0.001	0.000	0.025	0.013	0.001

Supplementary table S5: Relative abundance at genus level. The sampling sites reported: Natural Reserve Paititi (NRP); Santa Paula´s Farm (SP); Vivero Antoniucci (VA)

Sample			Genera												
**Code**	**Sampling site**	**Bumblebee Specie**	**Apibacter**	**Arsenophonus**	**Bifidobacterium**	**Gilliamella**	**Kluyvera**	**Lactobacillus**	**Pantoea**	**Pseudomonas**	**Rosenbergiella**	**Snodgrassella**	**Sodalis**	**Wolbachia**	**Others**
BB71	NRP	*B. bellicosus*	0.03	0.00	0.21	3.32	0.00	1.43	0.00	51.65	0.00	34.72	0.23	0.38	8.05
BB72	NRP	*B. bellicosus*	0.00	0.00	1.55	5.00	0.00	2.43	0.00	11.22	0.00	66.50	0.25	0.48	12.56
BB90	NRP	*B. bellicosus*	0.03	1.27	1.23	8.03	0.00	3.13	0.00	9.35	0.00	67.22	0.21	0.49	9.04
BB91	NRP	*B. bellicosus*	0.00	0.50	1.09	8.79	0.00	5.49	0.05	13.31	0.00	52.30	0.25	0.58	17.66
BB92	NRP	*B. bellicosus*	0.00	4.22	21.94	0.28	0.00	1.59	26.56	7.90	13.21	0.54	0.36	1.00	22.39
BB93	NRP	*B. bellicosus*	0.00	1.51	12.31	12.5	0.00	9.26	0.00	8.74	0.00	49.18	0.00	0.12	6.30
BB94	NRP	*B. bellicosus*	0.00	0.65	0.03	2.19	0.00	1.33	3.54	14.44	5.06	49.03	0.00	0.35	23.39
BB96	NRP	*B. bellicosus*	42.67	0.05	0.65	2.57	0.00	3.84	0.00	1.04	0.06	47.57	0.06	0.07	1.41
BP02	NRP	*B. pauloensis*	0.00	0.14	2.52	0.00	0.00	4.01	0.00	1.83	0.00	87.85	0.36	0.59	2.67
BP03	NRP	*B. pauloensis*	0.00	0.98	9.94	1.41	0.00	24.35	0.00	2.05	0.00	57.82	0.00	0.71	2.74
BP04	NRP	*B. pauloensis*	0.00	0.64	6.73	0.10	0.00	4.38	0.00	0.43	0.00	86.00	0.00	0.25	1.46
BP06	NRP	*B. pauloensis*	0.00	0.71	6.65	0.32	0.00	34.14	0.00	0.34	0.00	57.24	0.00	0.19	0.40
BP07	NRP	*B. pauloensis*	0.00	0.44	1.49	3.49	0.00	60.17	0.00	1.43	0.00	18.86	0.00	0.44	13.68
BP08	NRP	*B. pauloensis*	0.00	0.74	2.13	10.50	0.00	8.45	0.00	0.66	0.00	76.25	0.00	0.25	1.03
BP09	NRP	*B. pauloensis*	0.00	7.98	6.76	0.39	0.00	21.77	0.00	1.75	0.00	58.06	0.00	1.65	1.64
BP11	SP	*B. pauloensis*	4.02	0.12	18.92	6.44	0.00	10.08	0.00	4.36	0.00	24.81	1.30	2.63	27.32
BP13	SP	*B. pauloensis*	0.00	0.19	1.78	2.25	0.00	32.82	0.00	0.71	0.00	60.23	0.00	0.34	1.68
BP15	SP	*B. pauloensis*	2.42	0.49	2.11	11.10	0.00	35.64	0.00	0.52	0.12	45.09	0.23	0.39	1.88
BP16	SP	*B. pauloensis*	33.09	0.03	0.99	1.78	0.00	9.81	0.00	3.56	0.00	37.48	0.74	2.56	9.94
BP17	SP	*B. pauloensis*	0.00	0.22	0.00	0.04	19.27	41.64	0.00	1.23	0.11	0.12	0.40	1.25	35.72
BP18	SP	*B. pauloensis*	0.00	0.04	2.86	2.50	0.00	19.73	0.00	0.13	0.00	74.17	0.00	0.08	0.50
BP19	SP	*B. pauloensis*	25.55	0.03	1.66	4.40	0.00	7.65	0.00	0.72	0.00	53.28	0.00	0.03	6.68
BP20	VA	*B. pauloensis*	0.00	0.13	11.90	0.99	0.00	0.89	0.00	16.82	0.61	66.78	0.00	0.07	1.81
BP21	VA	*B. pauloensis*	0.00	0.00	1.81	6.76	0.00	12.58	0.00	0.36	0.00	75.85	0.00	0.00	2.63
BP22	VA	*B. pauloensis*	0.00	0.04	0.00	0.00	0.00	0.39	0.00	51.97	0.00	0.00	12.89	1.90	32.81
BP23	VA	*B. pauloensis*	4.03	0.35	0.05	1.50	0.00	20.59	0.08	22.57	0.00	4.24	6.49	15.5	24.54
BP24	VA	*B. pauloensis*	0.00	0.32	0.00	0.00	0.00	4.22	0.05	33.65	0.05	0.25	1.26	2.46	57.74
BP25	VA	*B. pauloensis*	0.00	0.62	3.91	2.69	0.00	17.00	0.09	14.23	0.00	26.56	4.82	7.68	22.41
BP26	VA	*B. pauloensis*	0.00	51.04	1.40	7.35	0.00	7.49	0.00	0.11	0.00	30.88	0.00	0.14	1.59
BP27	VA	*B. pauloensis*	0.02	6.35	19.32	5.58	0.00	29.41	0.00	0.30	0.00	37.49	0.00	0.22	1.30
BP28	VA	*B. pauloensis*	0.00	2.15	9.49	4.05	0.00	32.38	0.00	11.04	0.00	26.85	1.93	4.27	7.83

Supplementary table S6: Generalized Unifrac beta diversity, pairwise comparison of groups. The sampling sites reported: Natural Reserve Paititi (NRP); Santa Paula´s Farm (SP); Vivero Antoniucci (VA). p-values: * p<0.05; *** p<0.01.

Group_1	Group_2	Sample size	Permutations	pseudo-F	p-value	q-value
*B. bellicosus*RPN	*B. paulensis*RPN	15	999	2.161524	0.291	0.292
	*B. paulensis*SP	16	999	3.672724	0.031	0.093
	*B. paulensis*VA	17	999	2.625976	0.292	0.292
*B. paulensis*RPN	*B. paulensis*SP	15	999	11.745693	0.004 *	0.024
	*B. paulensis*VA	16	999	3.024549	0.207	0.292
*B. paulensis*SP	*B. paulensis*VA	17	999	4.755364	0.063	0.126

Supplementary table S7: Table summarizing the sample identification code and the NCBI Sequence Read Archive (SRA) accession number

Sample Code	Sampling site	Bumblebee Specie	NCBI accession number
BB71	NRP	*B. bellicosus*	SRR17715434
BB72	NRP	*B. bellicosus*	SRR17715423
BB90	NRP	*B. bellicosus*	SRR17715412
BB91	NRP	*B. bellicosus*	SRR17715401
BB92	NRP	*B. bellicosus*	SRR17715481
BB93	NRP	*B. bellicosus*	SRR17715470
BB94	NRP	*B. bellicosus*	SRR17715459
BB96	NRP	*B. bellicosus*	SRR17715448
BP02	NRP	*B. pauloensis*	SRR17715433
BP04	NRP	*B. pauloensis*	SRR17715431
BP06	NRP	*B. pauloensis*	SRR17715430
BP07	NRP	*B. pauloensis*	SRR17715429
BP08	NRP	*B. pauloensis*	SRR17715428
BP09	NRP	*B. pauloensis*	SRR17715427
BP11	SP	*B. pauloensis*	SRR17715425
BP13	SP	*B. pauloensis*	SRR17715422
BP15	SP	*B. pauloensis*	SRR17715421
BP16	SP	*B. pauloensis*	SRR17715420
BP17	SP	*B. pauloensis*	SRR17715419
BP18	SP	*B. pauloensis*	SRR17715418
BP19	SP	*B. pauloensis*	SRR17715417
BP20	VA	*B. pauloensis*	SRR17715416
BP21	VA	*B. pauloensis*	SRR17715415
BP22	VA	*B. pauloensis*	SRR17715414
BP23	VA	*B. pauloensis*	SRR17715413
BP24	VA	*B. pauloensis*	SRR17715411
BP25	VA	*B. pauloensis*	SRR17715410
BP26	VA	*B. pauloensis*	SRR17715409
BP27	VA	*B. pauloensis*	SRR17715408
BP28	VA	*B. pauloensis*	SRR17715407
BP03	NRP	*B. pauloensis*	SRR17715432
BP30	VA	*B. pauloensis*	SRR17715406

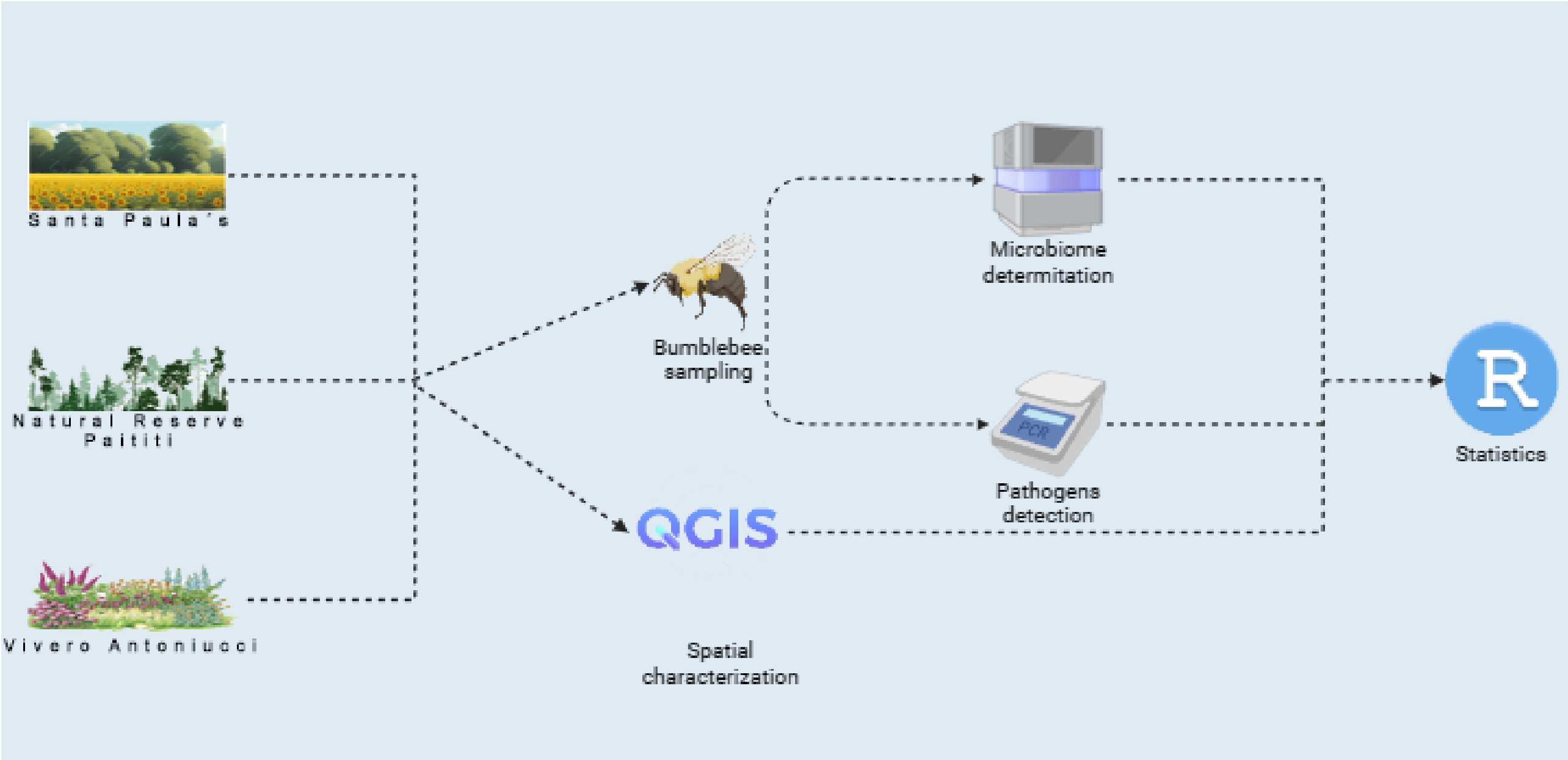

Supplementary figure S1: Workflow of the presented research illustrating the sampling sites and the analysis carried out.

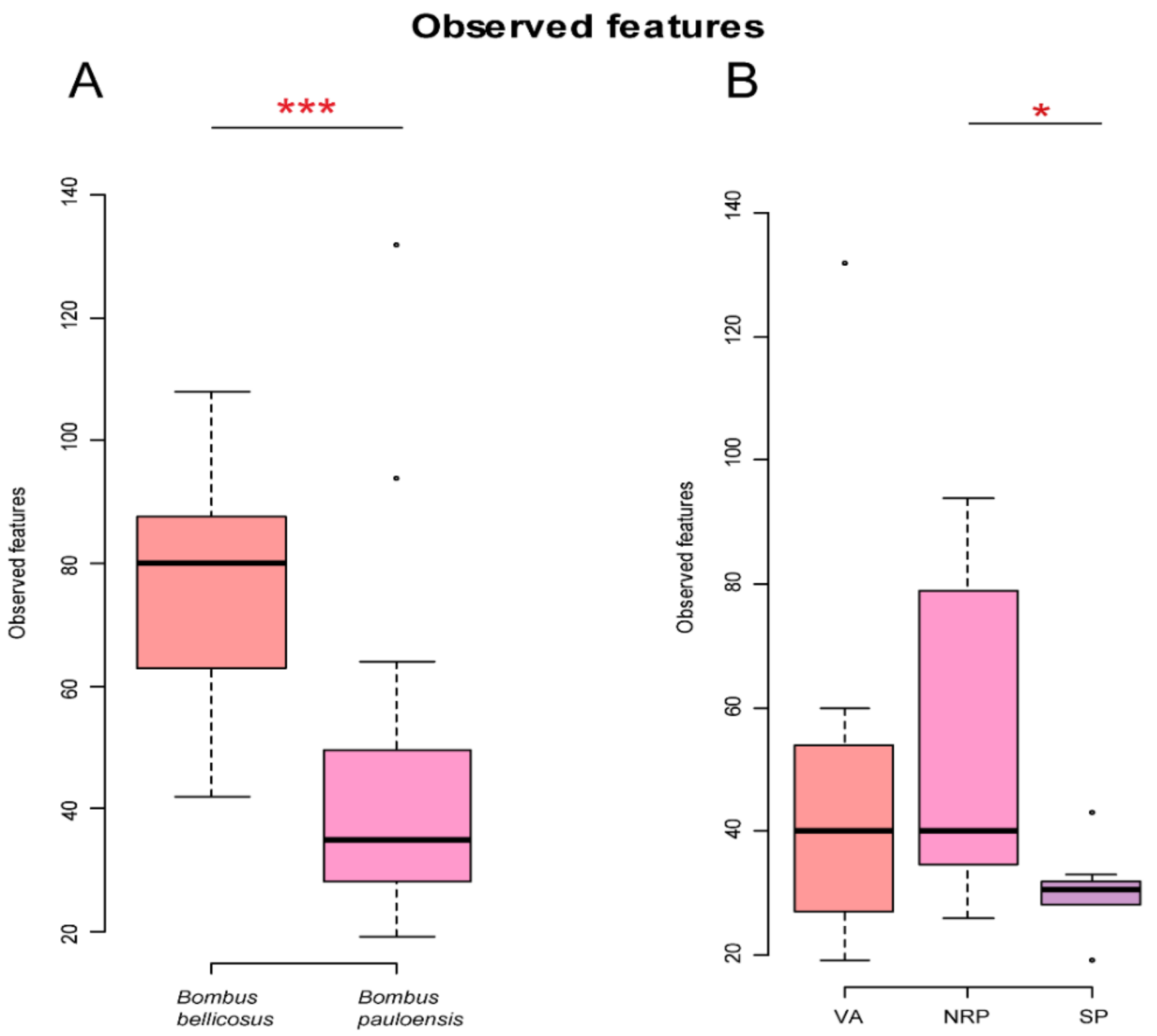

Supplementary figure S2: α-diversity indexes. A) Comparing *B. pauloensis* (n= 24) and *B. bellicosus* (n=8) with Observed features. B) Comparing the different sampling sites Vivero Antoniucci [VA] vs Reserva Natural Paititi [NRP] vs Santa Paula [SP] Observed features. p-values: * $p<0.05$; *** $p<0.01$.

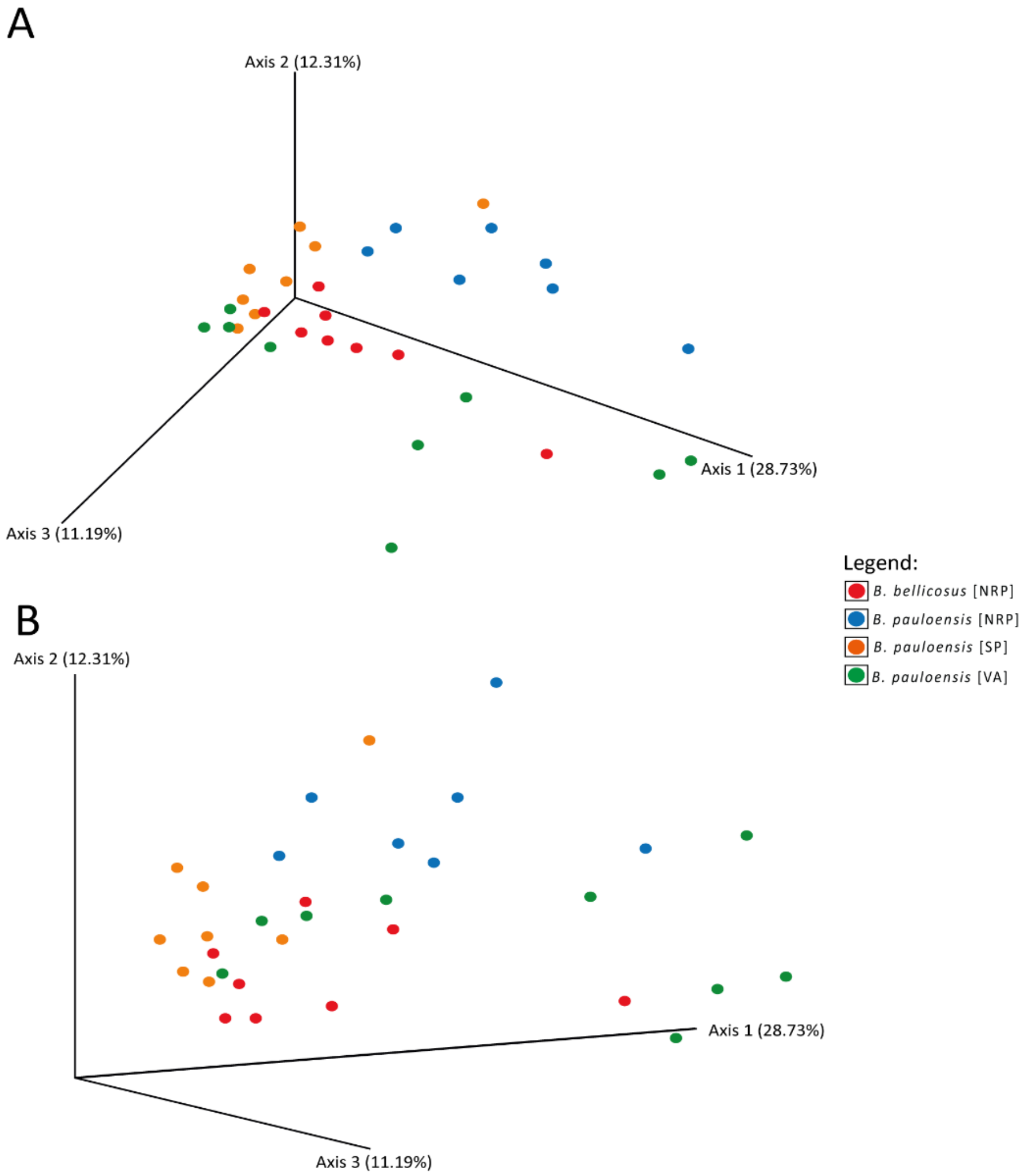

Supplementary figure S3: PCoA illustrating the Bray-Curtis β-Diversity. PCoA reports Bray-Curtis index for *B. pauloensis* sampled in Vivero Antoniucci [VA] (n= 9), Reserva Natural Paititi [NRP] (n=7) and Santa Paula [SP] (n=8), and for *B. bellicosus* sampled in [NRP]. Figure A) shows the samples distribution on axes 1-3, whereas, B) shows the same samples distribution on axes 1-2

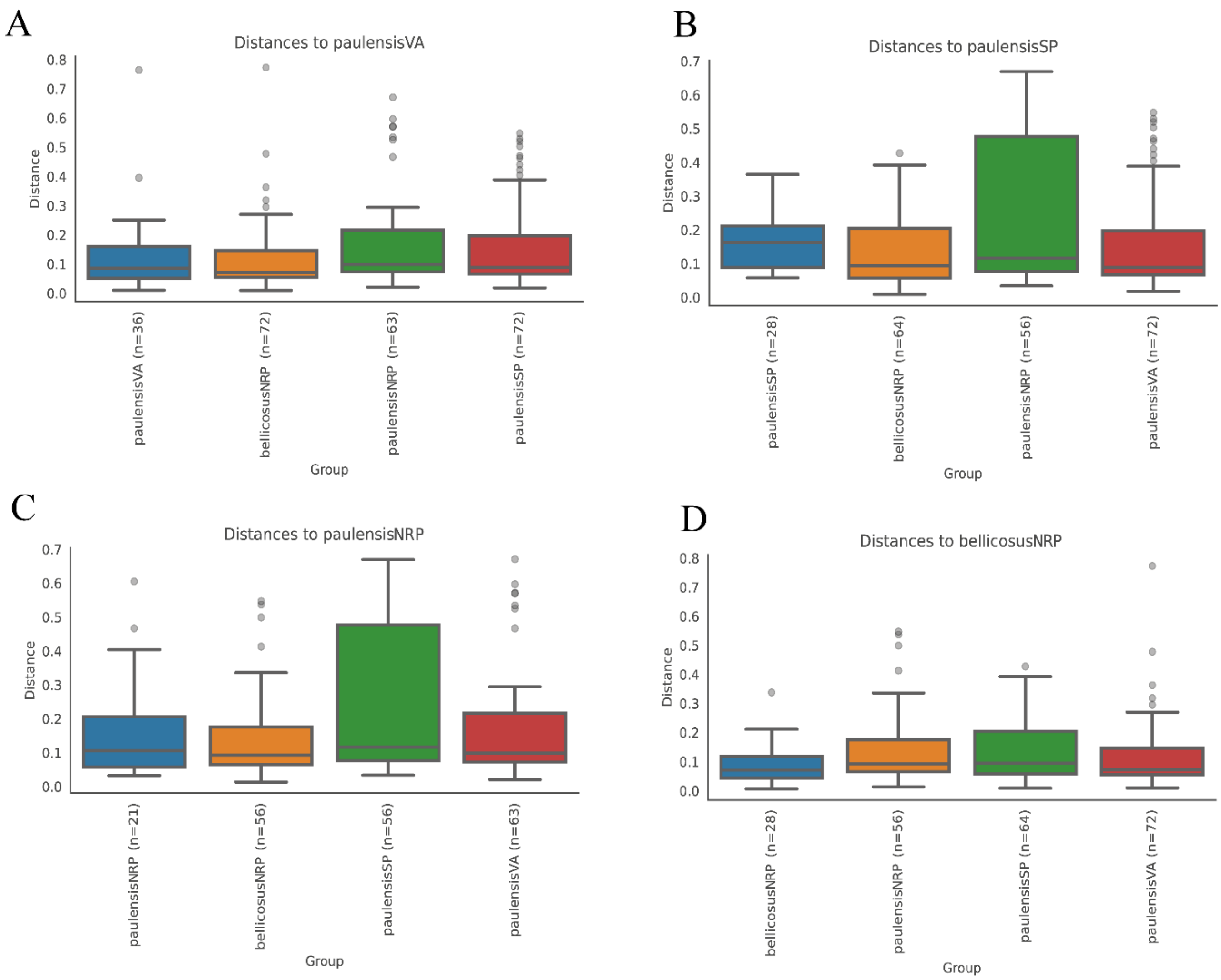

Supplementary figure S4: Generalized Unifrac. A) *B. paulensis* VA vs all sites and species. B) *B. paulensis* SP vs all sites and species.

A

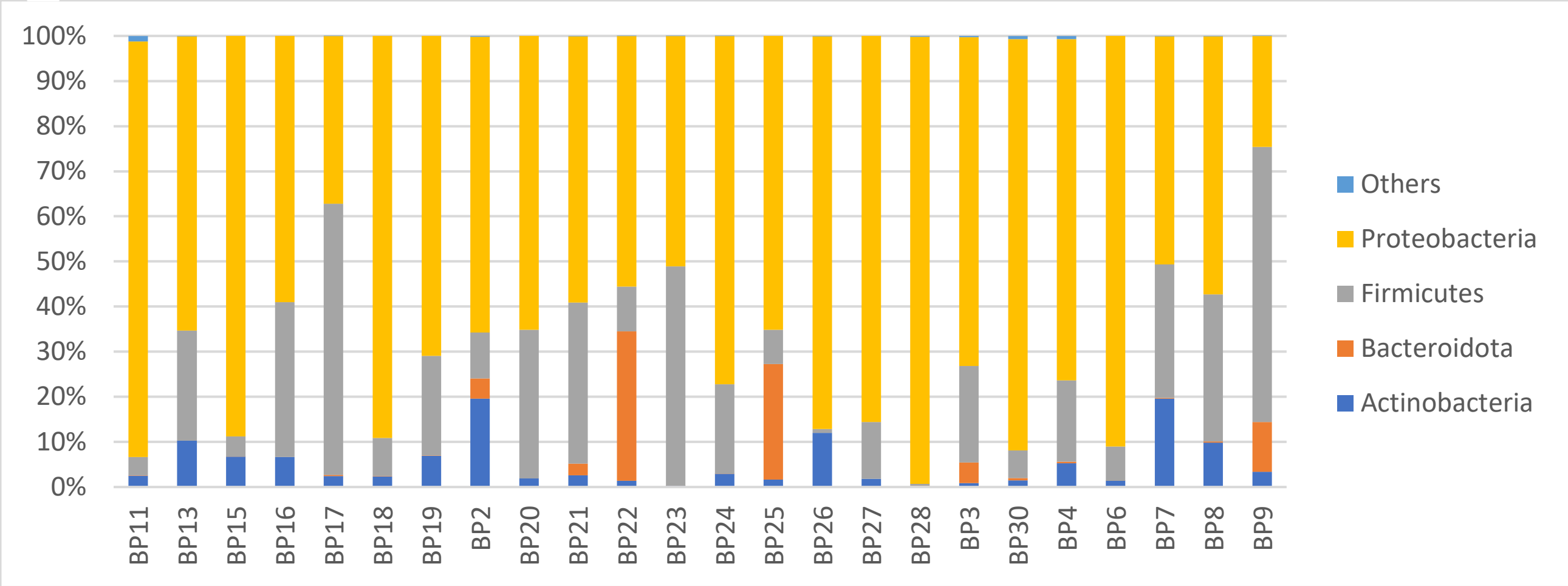

B

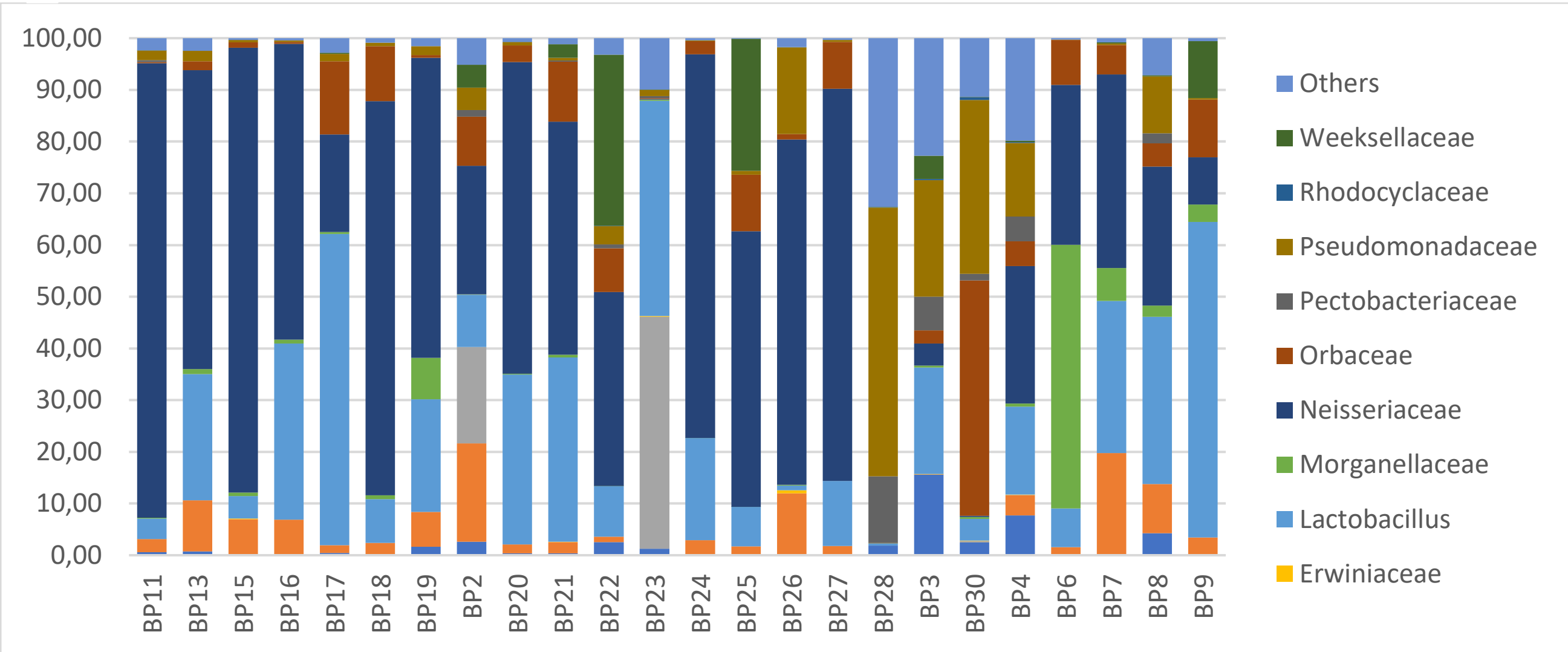

Supplementary figure S5: Diversity of the gut microbiota of *Bombus pauloensis*. Overlaid bar chart representing in percentage the composition of the microbiota (Y-axis) of each analyzed individual of *B. pauloensis* species (X-axis), for the different taxonomic levels studied, Phylum (A) and Family (B) level

A

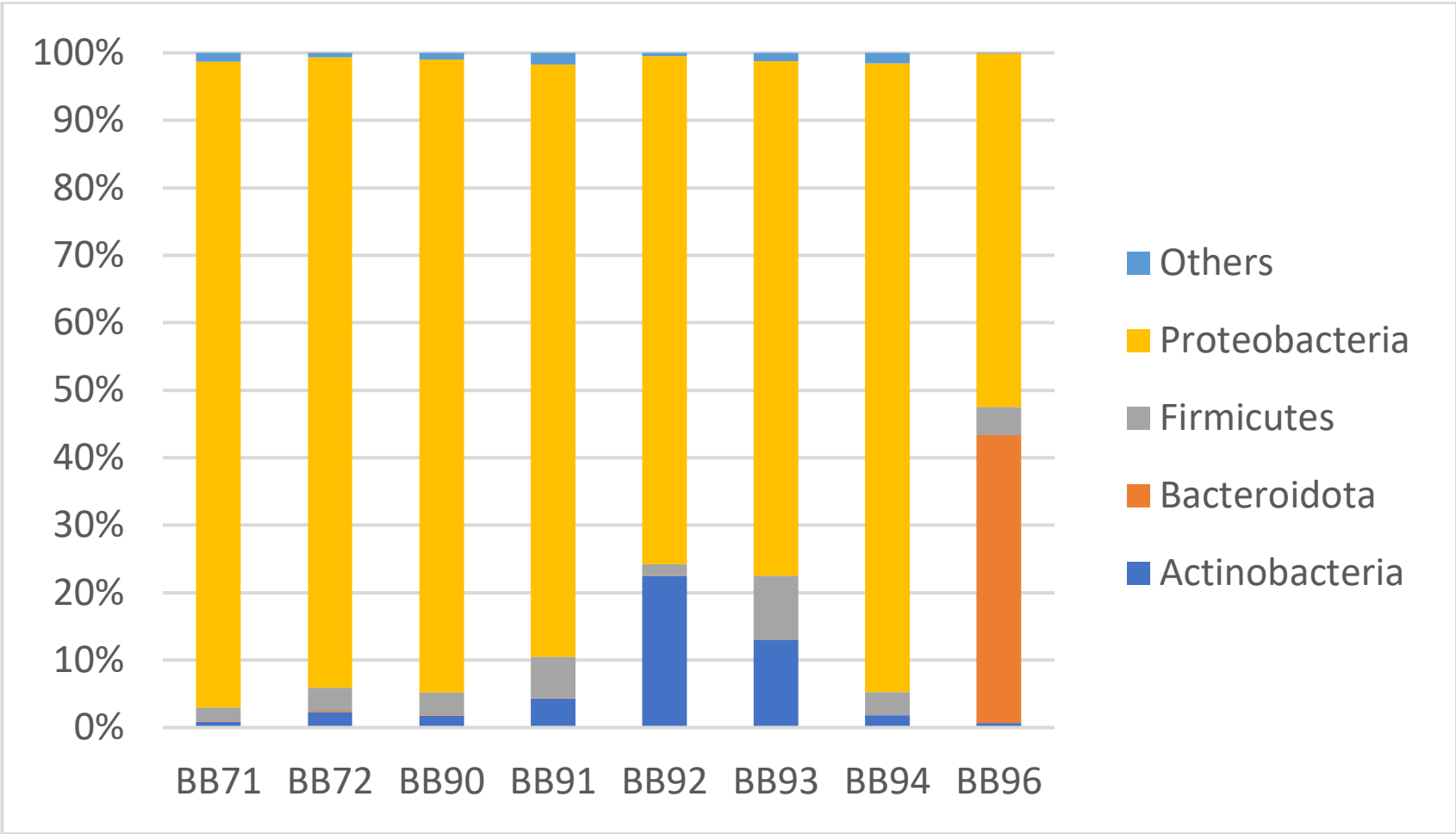

B

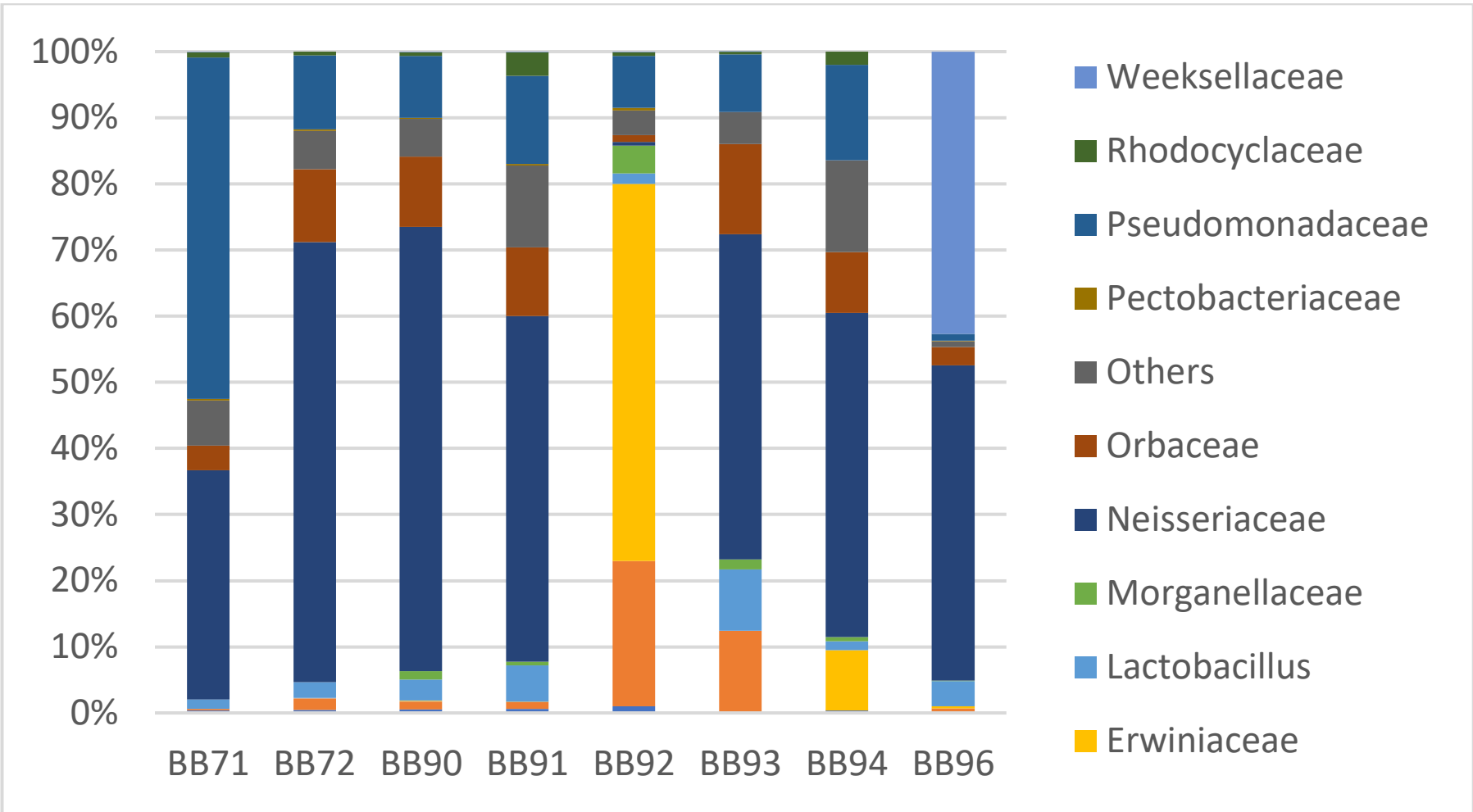

Supplementary figure S6: Diversity of the gut microbiota of *Bombus bellicosus*. Overlaid bar chart representing in percentage the composition of the microbiota (Y-axis) of each analyzed individual of *B. bellicosus* species (X-axis), for the different taxonomic levels studied, Phylum (A) and Family (B) level

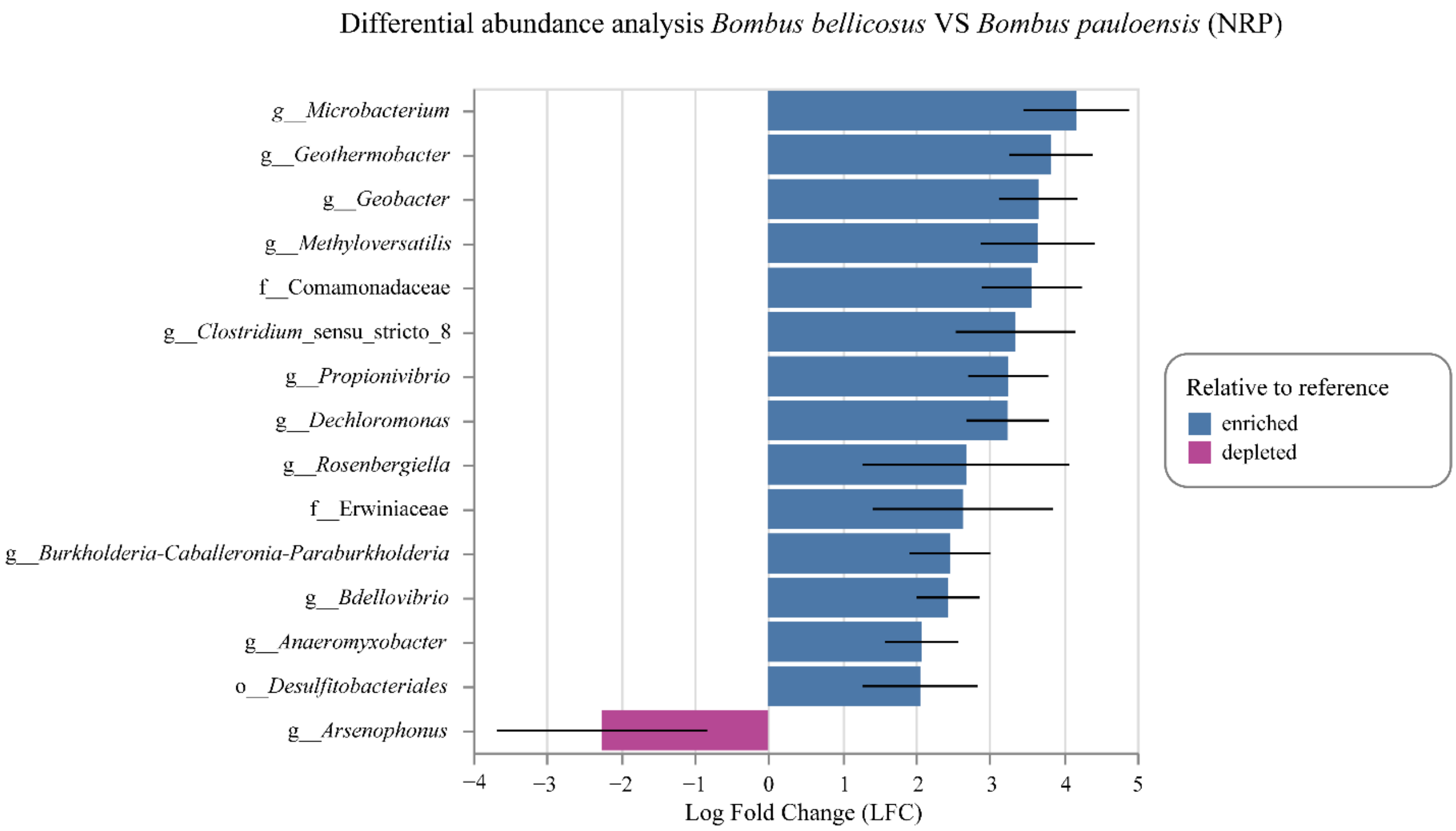

Supplementary figure S7: Differential abundance analysis (DAA) *Bombus bellicosus* compared to *Bombus pauloensis* in NRP sampling site. Only the differential values above Log Fold Change (LFC) of 2 are shown.

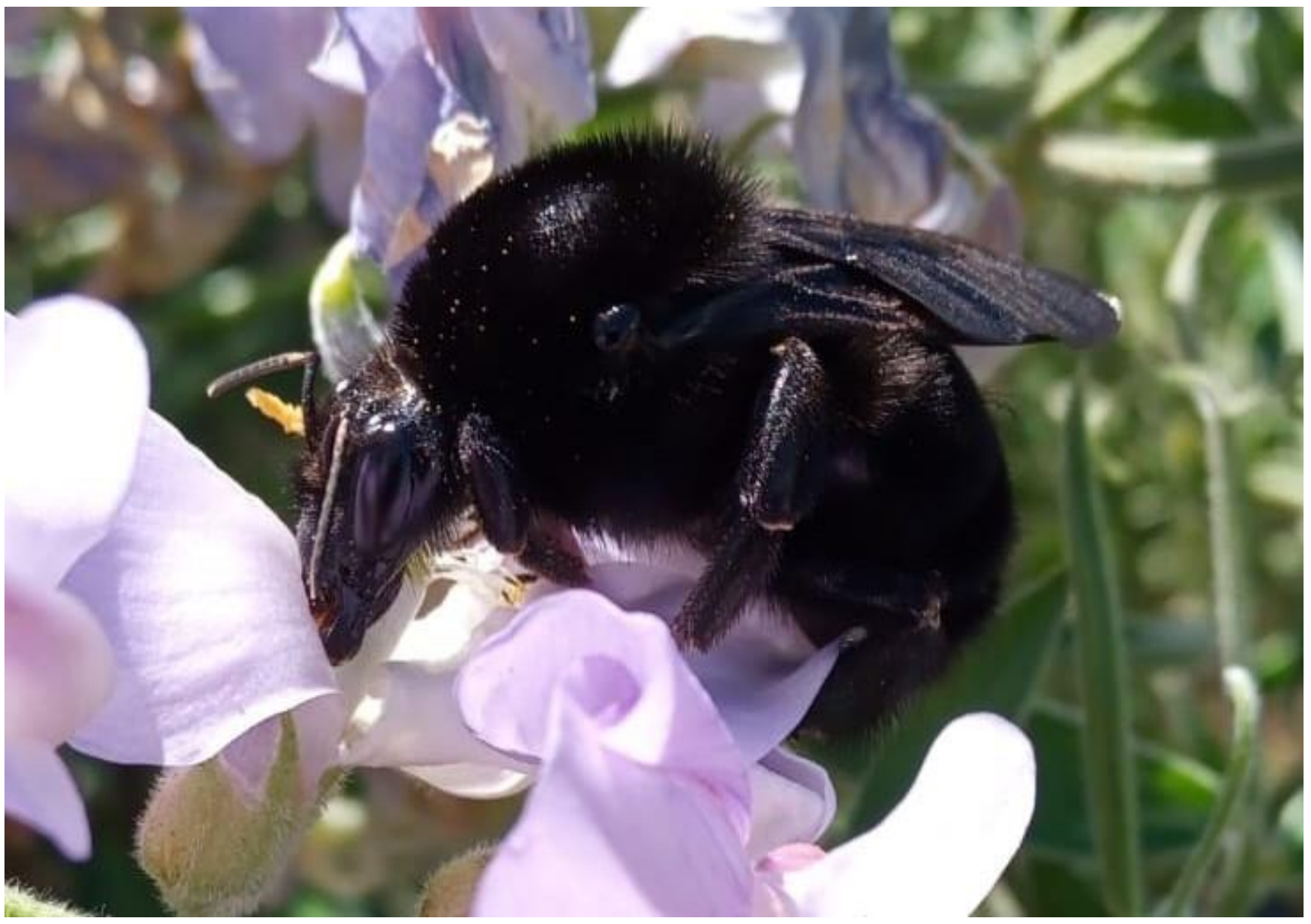

Supplementary figure S8: *Bombus pauloensis* foraging on a Leguminosae (Reserva Natural Paititi – Buenos Aires province December 2023)

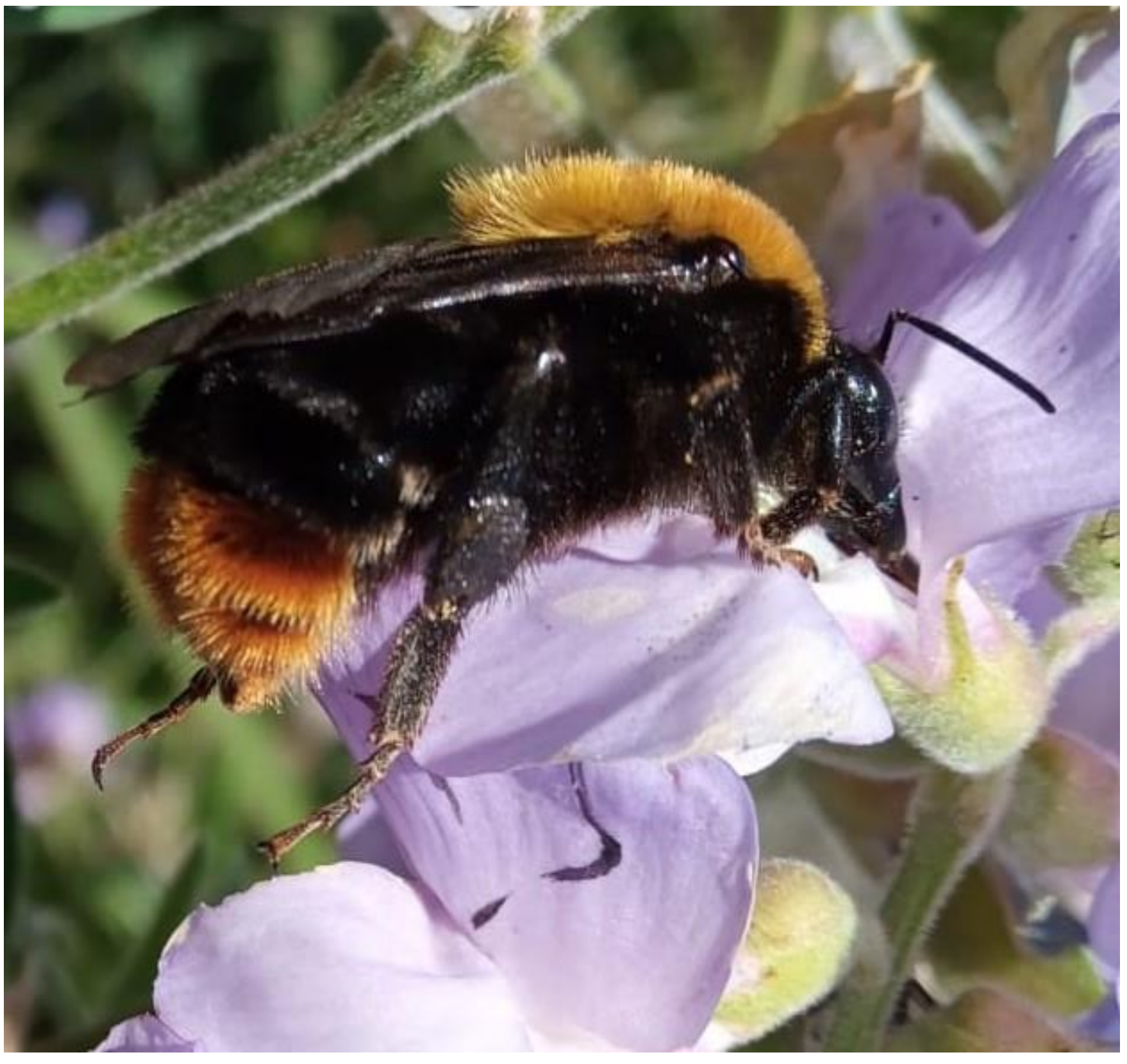

Supplementary figure S9: *Bombus bellicosus* foraging on a Leguminosae (Reserva Natural Paititi – Buenos Aires province, December 2023)

References:

Arismendi, N., Bruna, A., Zapata, N., & Vargas, M. (2016). PCR-specific detection of recently described Lotmaria passim (Trypanosomatidae) in Chilean apiaries. *Journal of invertebrate pathology*, *134*, 1-5. https://doi.org/10.1016/j.jip.2015.12.008

James, R. R., & Skinner, J. S. (2005). PCR diagnostic methods for Ascosphaera infections in bees. *Journal of Invertebrate Pathology*, *90*(2), 98-103. https://doi.org/10.1016/j.jip.2005.08.004

Martín-Hernández, R., Meana, A., Prieto, L., Salvador, A. M., Garrido-Bailón, E., & Higes, M. (2007). Outcome of colonization of Apis mellifera by Nosema ceranae. *Applied and environmental microbiology*, *73*(20), 6331-6338. https://doi.org/10.1128/AEM.00270-07

Huang, W. F., Skyrm, K., Ruiter, R., & Solter, L. (2015). Disease management in commercial bumble bee mass rearing, using production methods, multiplex PCR detection techniques, and regulatory assessment. *Journal of Apicultural Research*, *54*(5), 516-524. https://doi.org/10.1080/00218839.2016.1173352

Klee, J., Tay, W. T., & Paxton, R. J. (2006). Specific and sensitive detection of Nosema bombi (Microsporidia: Nosematidae) in bumble bees (Bombus spp.; Hymenoptera: Apidae) by PCR of partial rRNA gene sequences. *Journal of Invertebrate Pathology*, *91*(2), 98-104. https://doi.org/10.1016/j.jip.2005.10.012

Hartmann, U., Forsgren, E., Charrière, J. D., Neumann, P., & Gauthier, L. (2015). Dynamics of Apis mellifera filamentous virus (AmFV) infections in honey bees and relationships with other parasites. *Viruses*, *7*(5), 2654-2667. https://doi.org/10.3390/v7052654
